# *fosZ*, a novel plasmid-borne fosfomycin resistance gene in *Pseudomonas* species, especially carbapenem-resistant *Pseudomonas aeruginosa* isolates

**DOI:** 10.1128/aac.00750-25

**Published:** 2026-01-14

**Authors:** Youxing Shao, Xin Lan, Xuefei Zhang, Leilei Wang, Fan Yang, Minggui Wang, Qinglan Guo

**Affiliations:** 1 Institute of Antibiotics, Huashan Hospital, Fudan University 198171, Shanghai, People's Republic of China; 2 Key Laboratory of Clinical Pharmacology of Antibiotics, National Heath Commission of the People's Republic of China, Shanghai, China; 3 Department of Clinical Laboratory, Affiliated Cancer Hospital of Zhengzhou University 377327 , Zhengzhou, Henan, China; Universita degli Studi di Roma "La Sapienza", Rome, Italy

**Keywords:** *fosZ*, fosfomycin, IS*Pa75*, plasmid, *Pseudomonas aeruginosa*

## Abstract

A novel fosfomycin-resistant glutathione S-transferase (FR-GST) gene, *fosZ*, was investigated, and its structural characteristics were characterized in *Pseudomonas* species. The *fosZ* gene was cloned and expressed in *P. aeruginosa* PAO1 and HS355, where it displayed reduced susceptibility to fosfomycin (8- to 64-fold) and its inhibitor sodium phosphonoformate (PPF). FosZ shares less than 70% amino acid identity with known FosA proteins. Bioinformatics analyzes revealed that *fosZ* was a transposable passenger gene within IS*Pa75*, likely captured from *Pseudomonas* species. A total of 159 *fosZ*-bearing *Pseudomonas* strains were identified in GenBank over the past 22 years, sharing ten target site duplications (TSDs) associated with IS*Pa75*. Among them, 34 strains were fully sequenced. IS*Pa75-fosZ* was found on at least two chromosomes and 33 plasmids from four incompatibility groups, including the megaplasmids IncP-2 (24/33) and Inc_pJBCL41_ (8/33), which also carried carbapenem and other antibiotic resistance genes. The most common TSDs were TSD6 (82/156) and TSD10 (48/156), predominantly in carbapenem-resistant *P. aeruginosa* of various sequence types, especially ST1076, ST1418, and ST463. Mobilization of IS*Pa75* between plasmid and chromosome was observed in *P. aeruginosa* MAS152. Structure prediction and analysis of FR-GST proteins revealed distinct features in the dimer interface loop. In the FosZ structure, an expanded K^+^-binding loop caused a deviation at residue S95, which participated in binding both fosfomycin and PPF. In conclusion, *fosZ* encodes a distinct FR-GST exhibiting reduced susceptibility to inhibition by PPF and has been acquired by transferable IS*Pa75* on multidrug-resistant megaplasmids, disseminating fosfomycin resistance in *Pseudomonas* species, particularly across China.

## INTRODUCTION

Multidrug-resistant *Pseudomonas aeruginosa* (MDR-PA) poses a serious public health concern. The limited therapeutic options for combating MDR-PA and extensively drug-resistant *P. aeruginosa* (XDR-PA) have reignited interest in older, underutilized drugs like fosfomycin (1, 2). Fosfomycin has potent *in vitro* antibacterial activity against a broad spectrum, has led to its reemergence in combination with other antimicrobials for treating MDR-PA bacterial infections (3, 4). However, fosfomycin use encounters challenges from inherent and acquired resistance mechanisms. Fosfomycin resistance mechanisms in *P. aeruginosa* encompass (5) (i) the existence of recycling pathways bypassing *de novo* peptidoglycan biosynthesis, (ii) mutations in the fosfomycin transporter GlpT, and (iii) chromosomal fosfomycin-modifying enzymes, including FosA^PA^, FosE, FosF, and FosH.

Fosfomycin-modifying enzymes include three types of metalloenzymes (1, 2, 5): Mn^2+^- and K^+^-dependent glutathione S-transferase (e.g. FosA), Mg^2+^-dependent bacillithiol transferase (e.g., FosB), Mn^2+^-dependent hydrolase FosX, and fosfomycin kinases. Glutathione S-transferases, represented by FosA enzymes, are frequently found in chromosomes of Gram-negative species (6). Eleven variants of FosA (FosA1 to FosA11) have been characterized (7–12) and have been acquired by transposons and/or plasmids mostly in MDR *Enterobacterales* (Table S1). In *P. aeruginosa*, *fosA* homologs are present in nearly 99% of the 2,257 surveyed genomes (6). Several other acquired glutathione-S-transferase genes, like *fosC2*, *fosK*, and *fosL1*, have also been identified in Gram-negative species (13, 14).

Sodium phosphonoformate (PPF), approved for clinical use in treating cytomegalovirus infection and acyclovir-resistant herpes simplex virus infection, is a competitive inhibitor of glutathione S-transferase (15). PPF has been effective in detecting the production of glutathione S-transferase responsible for fosfomycin resistance (FR-GST), such as FosA3, FosA4, and FosC2 in *Escherichia coli* (16), and chromosomal *fosA* from multiple Gram-negative species, including *P. aeruginosa* (6).

In our prior studies, we reported a carbapenem-resistant *P. aeruginosa* (CRPA) isolate HS17-127 (17, 18), which co-carried the metallo-β-lactamase (MBL) genes *bla*_AFM-1_ and *bla*_IMP-45_ within an MDR transposon situated on a conjugative IncP-2 megaplasmid, pHS17-127. In this study, we identified a new FR-GST gene, named *fosZ*, located on pHS17-127, encoding FosZ enzyme, which is surprisingly not inhibited by PPF. This study represents the first exploration of plasmid-borne fosfomycin-modifying enzyme in *P. aeruginosa*.

## MATERIALS AND METHODS

### Bacterial isolates and antimicrobial susceptibility testing

The CRPA isolate HS17-127 and its transconjugant (18), along with the fosfomycin-susceptible *P. aeruginosa* isolate HS355 (19) and fosfomycin-resistant *Enterobacter cloacae* 19-4074 (*fosA3-*positive), were obtained in our previous study. Antimicrobial susceptibility testing for fosfomycin was performed using the agar dilution method, following 2024 Clinical and Laboratory Standards Institute (CLSI) recommendations (20). Glutathione S-transferase activity inhibition was examined using PPF, as previously described (6, 8).

### Cloning experiments

The complete coding sequence of *fosZ* (411 bp) was cloned into *Escherichia-Pseudomonas* shuttle vector pUCP19 (Shanghai Bioresource Collection Center, China). To confirm the role of the promoter region in FosZ expression, which includes the full *fosZ* gene and a 300 bp upstream region with its native promoter, was also cloned into pUCP19. The recombinant plasmids were transformed into *P. aeruginosa* PAO1 and HS355, as well as *E. coli* DH5α. The specific primers are detailed in Table S2.

### Bioinformatic analysis

The phylogenetic tree was built using the Neighbor-Joining method with MEGA-X (21) and visualized with ChiPlot (22). Antimicrobial resistance genes were screened using ResFinder (https://genepi.food.dtu.dk/resfinder), and mobile genetic elements (MGEs) were identified using ISfinder and BLASTN (https://blast.ncbi.nlm.nih.gov/Blast.cgi). BLASTP and BLASTN tools were used to search for homologues of FosZ and its encoding gene *fosZ*.

### Protein structure prediction and study

The protein structures of FR-GSTs were predicted using ColabFold (https://github.com/sokrypton/ColabFold) (23), and these coordinates were visualized using PyMOL.

## RESULTS

### Identification of *fosZ* on pHS17-127

**Fig 1:**
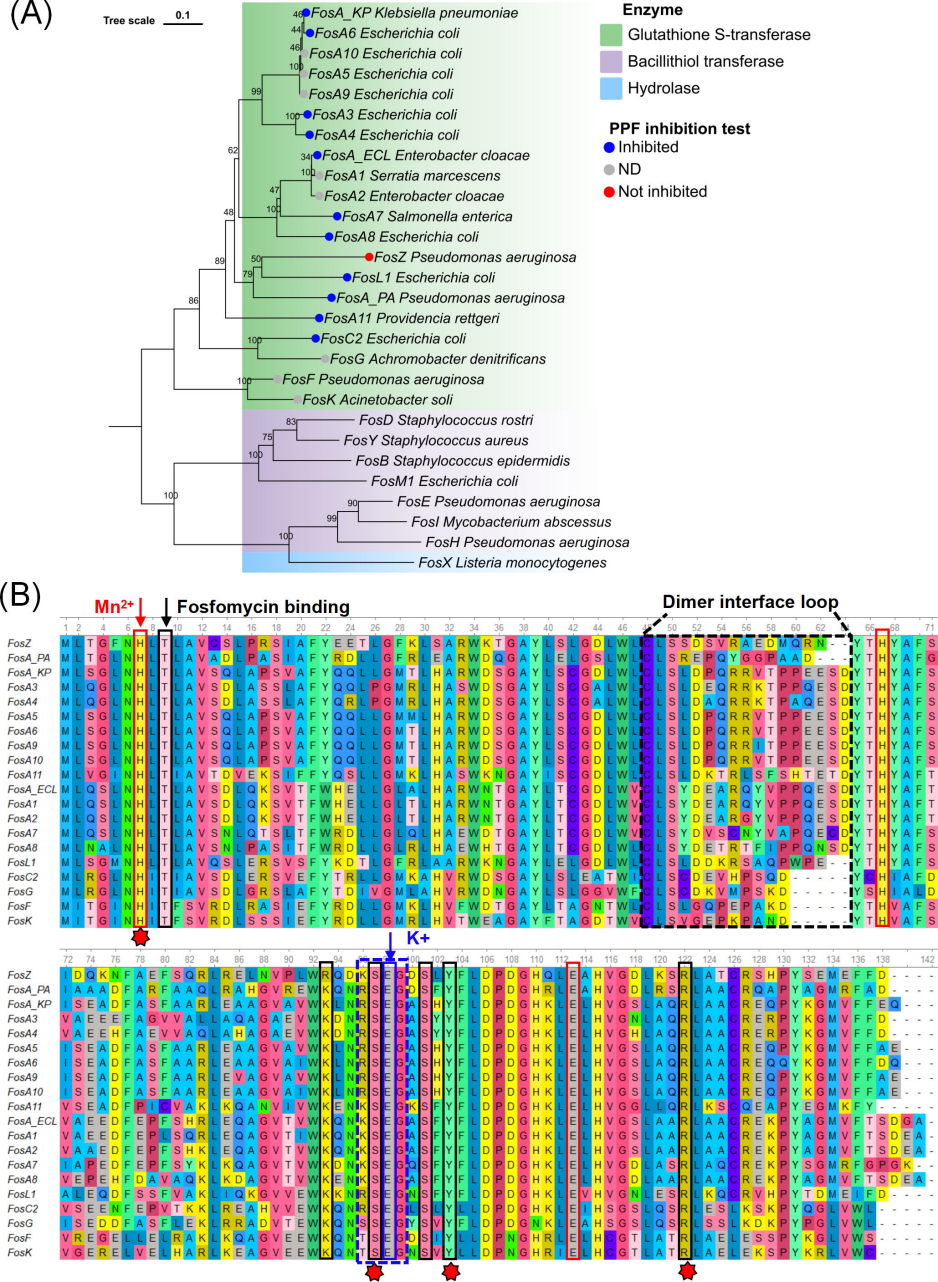
Phylogenetic reconstruction of known fosfomycin-modifying enzymes and structural alignment of FR-GSTs. (**A**) Phylogenetic tree of fosfomycin-modifying enzymes constructed using neighbor-joining algorithm in MEGAX. Branch lengths are scaled by 1,000 bootstrap replications. FosA with superscript notation indicates chromosomal-encoded FosA from different species: *P. aeruginosa* (PA), *E. cloacae* (ECL), and (*K. pneumoniae* (KP). (**B**) Amino acid sequence alignment of FR-GSTs using ClustalW. The residue positions in the sequence alignment were calibrated based on FosA^KP^. Residues associated with fosfomycin binding, Mn^2+^ coordination, and K^+^ coordination are highlighted with black, red, and blue colored boxes, respectively (25). Black and blue dashed boxes bracket amino acids of the dimer interface loop and K^+^-binding loop, respectively. The asterisk symbol indicates four residue positions where PPF competes with fosfomycin for binding inhibition (26).

CRPA isolate HS17-127 exhibited a MDR profile (18). Both HS17-127 and its transconjugant (TcHS17-127) displayed a fosfomycin minimum inhibitory concentration (MIC) of 128 mg/L (Table 1). We found a 411 bp open reading frame (ORF) on pHS17-127, annotated as a putative glutathione S-transferase. This ORF encoded a protein of 136 amino acids, sharing 43% to 62% amino acid identity with other identified FR-GSTs (Fig. S1). We designated this new gene as *fosZ*, following the latest *fosY* nomenclature (24). Phylogenetic analysis of amino acid sequences of FosZ and other FR-GSTs revealed that FosZ was distantly related to the FosA family (less than 70%) and represented a distinct FR-GST cluster (Fig. 1A). FosZ showed the highest amino acid homology with chromosome-encoded FosA^PA^ (62%) in *P. aeruginosa*, and FosL1 (60%) in *E. coli* (Fig. S1, Fig. 1A). Amino acid alignment of FosZ with other FR-GSTs revealed conserved residues for Mn^2+^ and K^+^ cations, along with a K91R substitution in the fosfomycin-binding site (Fig. 1B).

**TABLE 1:** Fosfomycin susceptibility of *P. aeruginosa* and *E. coli* strains harboring *fosZ*

Isolate	Fosfomycin MIC (mg/L)^*b*^			
	Without PPF	PPF (500 mg/L)	PPF (1,000 mg/L)	PPF (2,000 mg/L)
*P. aeruginosa* HS17-127	128	128	128	128
TcHS17-127*^a^*	128	128	128	64
*P. aeruginosa* PAO1(*fosA*^PA^)	64	16	16	16
*P. aeruginosa* PAO1 (pUCP19)	64	16	16	16
*P. aeruginosa* PAO1 (pUCP19-*fosZ*)	256	256	256	256
*P. aeruginosa* PAO1 (pUCP19-pro-*fosZ*)	512	512	512	512
*P. aeruginosa* HS355	8	8	8	8
*P. aeruginosa* HS355 (pUCP19-*fosZ*)	512	512	512	512
*P. aeruginosa* HS355 (pUCP19-pro-*fosZ*)	512	512	512	512
*E. cloacae* 19-4074 (*fosA3*)^*c*^	1024	256	256	256
*E. coli* DH5α	0.5	1	1	ND^*d*^
*E. coli* DH5α (pUCP19-*fosZ*)	0.5	1	1	ND
*E. coli* DH5α (pUCP19-pro-*fosZ*)	2	2	4	ND

^*a*^ TcHS17-127 is the *P. aeruginosa* PAO1 transconjugant harboring plasmid pHS17-127 from isolate HS17-127.

^*b*^ Sodium phosphonoformate (PPF) was added to Mueller-Hinton agar at 500 mg/L, 1000 mg/L, and 2000 mg/L, respectively.

^*c*^ A *fosA3*-positive strain.

^*d*^ ND, not determined.

### FosZ enzyme confers fosfomycin resistance and escapes inhibition by PPF in *P. aeruginosa*

To investigate the functionality of *fosZ*, both the gene itself and the gene along with its native promoter region were cloned and subsequently transformed into *P. aeruginosa* PAO1, HS355 (19) and *E. coli* DH5α. HS355 was a clinical MDR isolate that was susceptible to fosfomycin. The MIC of fosfomycin for the PAO1 transformant (pUCP19-*fosZ*) was 128 mg/L, while the PAO1 transformant with the native promoter (pUCP19-pro-*fosZ*) exhibited a fosfomycin MIC of 512 mg/L, an eightfold increase compared to PAO1 carrying the empty vector (Table 1). HS355 transformants (pUCP19-*fosZ* and pUCP19-pro-*fosZ*) showed a fosfomycin MIC of 512 mg/L, representing a 64-fold increase relative to HS355 (MIC 8 mg/L) (Table 1). *E. coli* DH5α transformant (pUCP19-pro-*fosZ*) demonstrated a more moderate fourfold elevation in fosfomycin MIC (Table 1). These results imply that the FosZ enzyme can reduce fosfomycin susceptibility in *P. aeruginosa*.

The glutathione S-transferase inhibitor PPF was tested for its inhibitory effect on FosZ, FosA^PA^ in PAO1, and FosA3 in *E. cloacae* 19-4074. PPF reduced the fosfomycin MIC in PAO1 from 64 to 16 mg/L and in *E. cloacae* 19-4074 from 1,024 to 256 mg/L. In contrast, none of the tested *fosZ*-bearing strains (clinical isolate, transconjugant, and transformants) exhibited any change in fosfomycin MIC (Table 1).

### Origin of FosZ and genetic context of *fosZ*

BLASTP results revealed that FosZ was identical to a vicinal oxygen chelate (VOC) family protein (WP_065760318.1) of *Pseudomonas* species (27) and related to 45 VOC proteins with identities between 70.6% and 89.7% in the Nonredundant Protein Database as of 9 Oct 2024 (Table S3). Most of them (44/45, 97.9%) identified in *Pseudomonas* species (Table S3). Two FosZ-like proteins, FosZ-like_PAGU2196_ (WP_238216096.1) and FosZ-like _LP_7_YM_ (WP_133752033.1) recovered from *Pseudomonas* sp. PAGU 2196 and LP_7_YM were closest to FosZ with identities of 89.7% and 87.5%. The *fosZ* gene shared 89.8% and 84.4% nucleotide similarities with these two FosZ-like-encoding genes, respectively. No mobile genetic elements were found associated with the two *fosZ*-like genes by ISfinder. In contrast, we found an insertion sequence (IS) of IS*66* family, IS*Pa75* (2,996 bp in length) had entrapped *fosZ* in pHS17-127. There are 110 and 138 FosZ-encoding sequences in the NCBI Reference Sequence (RefSeq) project and International Nucleotide Sequence Database Collaboration (INSDC) (https://www.ncbi.nlm.nih.gov/ipg/WP_065760318.1). After eliminating duplicate strains, 159 *fosZ*-bearing *Pseudomonas* strains of various species were obtained, including *P. aeruginosa* (*n* = 145), *P. putida* (*n* = 6), *P. fulva* (*n* = 5), and one each of *P. asiatica*, *P. oleovorans,* and *P. defluvii* (Table S4). All the *fosZ* genes retrieved were located in IS*Pa75* with identical sequences.

**Fig 2:**
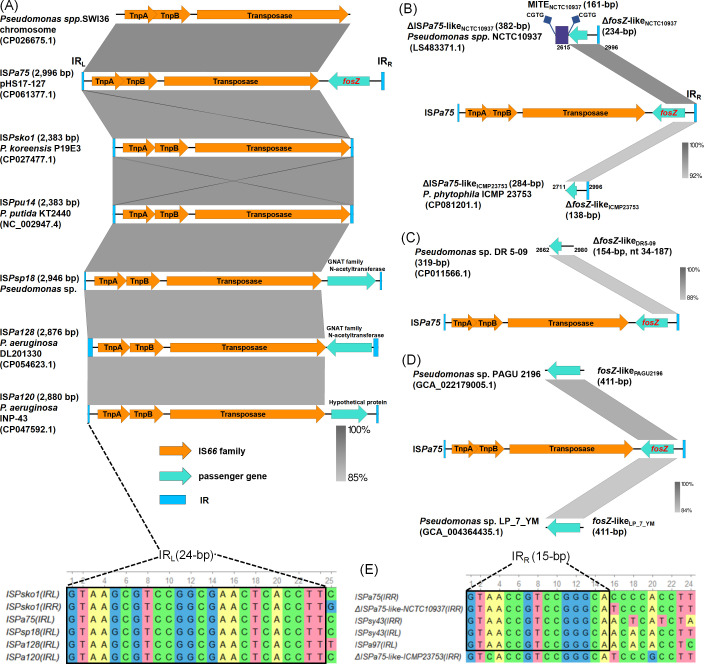
Comparison of genetic context of *fosZ* with representative IS*66* family members. (**A**) Comparison of left side of IS*Pa75* with IS*Pa75*-like on the chromosome of *Pseudomonas* sp. SWI36, IS*Psko1*, IS*Ppu14*, IS*Psp18*, IS*Pa128*, and IS*Pa120*. Below is the nucleotide sequence alignment of the IR_L_ and IR_R_ of IS*Psko1*, and the IR_L_ of IS*Pa75*, IS*Psp18*, IS*Pa128,* and IS*Pa120*. (**B**) Comparison of IS*Pa75* with two remnants of ΔIS*Pa75*-like_NCTC10937_ and ΔIS*Pa75*-like_ICMP23753_. The flanking direct repeats (CGTG) of MITE_NCTC10937_ are indicated by the paired squares. (**C**) Comparative analysis of IS*Pa75* with ΔIS*Pa75*-like_DR5-09_, a 319-bp fragment containing Δ*fosZ*-like_DR5-09_ in the chromosome of *Pseudomonas* sp. DR 5-09. (**D**) Comparative analysis of *fosZ* with *fosZ*-like_PAGU2196_ and *fosZ*-like_LP_7_YM_, with short flanking sequences. (**E**) Nucleotide sequence alignment of the IR_R_ of IS*Pa75*, ΔIS*Pa75*-like_NCTC10937_, IS*Psy43*, and ΔIS*Pa75*-like_ICMP23753_ and the IR_L_ of IS*Psy43* and IS*Pa97*.

Like classic IS*66* family members (*c*IS*66*s), IS*Pa75* encompassed three ORFs encoding proteins: IS*66* TnpA, IS*66* TnpB, and a transposase harboring a potential DDE catalytic triad motif (Fig. 2A). Beyond that, however, IS*Pa75* integrated a passenger resistance gene, *fosZ,* downstream of the DDE transposase. Searching in the ISfinder database, we found 254 IS*66* family members, and 25 were discovered in *Pseudomonas* species (Table S5). Seven out of 25 carried passenger genes, referred to as *t*IS66 (denoting a frontier between ISs and transposons) (28). IS*Pa75* shared 93% nucleotide identity with *c*IS*66* IS*Psk01*, 91% with *c*IS*66* IS*Ppu14*, 93% with *t*IS*66* IS*Psp18*, 90% with *t*IS*66* IS*Pa128*, and 90% with *t*IS*66* IS*Pa120* on the left side (2263 to 2312 bp), all of which were from *Pseudomonas* species (Fig. 2A). The IS*Psko1* had 24-bp identical sequences at terminal inverted repeat left and right (IR_L_ and IR_R_, GTAAGCGTCCGGCGAACTCACCTT), which are same to those at IR_L_ of IS*Pa75*, IS*Psp18*, IS*Pa120,* and IS*Pa128* (Fig. 2A).

Besides, BLASTN analysis in the core nucleotide database (core_nt) revealed a putative IS*66* family member located in the chromosome of *Pseudomonas* sp. SWI36 (CP026675.1) with 94% nucleotide similarity with IS*Pa75* (nt 14 to 2333, Fig. 2A). We also found two remnants of IS*Pa75*-like elements at the right side, named ΔIS*Pa75*-like_NCTC10937_ and ΔIS*Pa75*-like_ICMP23753_ in the chromosomes of *Paucimonas lemoignei* strain NCTC10937 (LS483371.1, which was reidentified as a potential new species of *Pseudomonas* sp. by JSpeciesWS, https://jspecies.ribohost.com/jspeciesws/#home), and *P. phytophila* strain ICMP 23753 (CP081201.1). The 382-bp-length ΔIS*Pa75*-like_NCTC10937_ had a nucleotide similarity of 96.6% at nt 2,615 to 2,996, containing 234-bp Δ*fosZ*-like gene (98.3% similarity) and 148-bp right-side sequence of ΔIS*Pa75*-like (93.9% similarity) (Fig. 2B). The 284-bp ΔIS*Pa75*-like_ICMP23753_ had a nucleotide similarity of 92.0% at nt 2,711 to 2,996, containing 138-bp Δ*fosZ*-like gene (92.8% similarity) and 146-bp right-end ΔIS*Pa75*-like (91.8% similarity) (Fig. 2B). We discovered a new miniature inverted-repeat transposable element (MITE), MITE_NCTC10937_ of 161-bp length which truncated the *fosZ*-like_NCTC10937_ gene and generated 4-bp DR (CGTG) (Fig. 2B). There also existed a fragment of 319-bp length in the chromosome of *Pseudomonas* sp. DR 5-09 (CP011566.1) sharing a similarity of 88.1% with IS*Pa75* at nt 2662 to 2980, including 154-bp-length of Δ*fosZ*-like gene (89.6% similarity, nt 34-187) (Fig. 2C). The *fosZ*-like_PAGU2196_ and *fosZ*-like_LP_7_YM_ genes with short flanking sequences (14 to 51 bp) had similarities of 88.5% and 84.4% to *fosZ* and its surrounding sequences (Fig. 2D).

The IR_R_ of IS*Pa75* was similar to the IR_R_ of ΔIS*Pa75*-like_NCTC10937_ and the IR_L_ of IS*Psy43* (*t*IS*66*) and IS*Pa97*, with similarities of 95.8% (23/24), 91.7% (22/24), and 87.5% (21/24) respectively (Fig. 2E). The 15-bp terminal IR (GTAACCGTCCGGGCA) was highly conserved (100%, 15/15) in the IR_R_s of IS*Pa75*, ΔIS*Pa75*-like_NCTC10937_ and IS*Psy43*, and IR_L_s of IS*Psy43* and IS*Pa97* (Fig. 2E). It is supposed that *fosZ* was derived from an unknown *Pseudomonas* species and entrapped by IS*66s* with terminals closely related to those of IS*Psko1* and IS*Psy43*.

**Fig 3:**
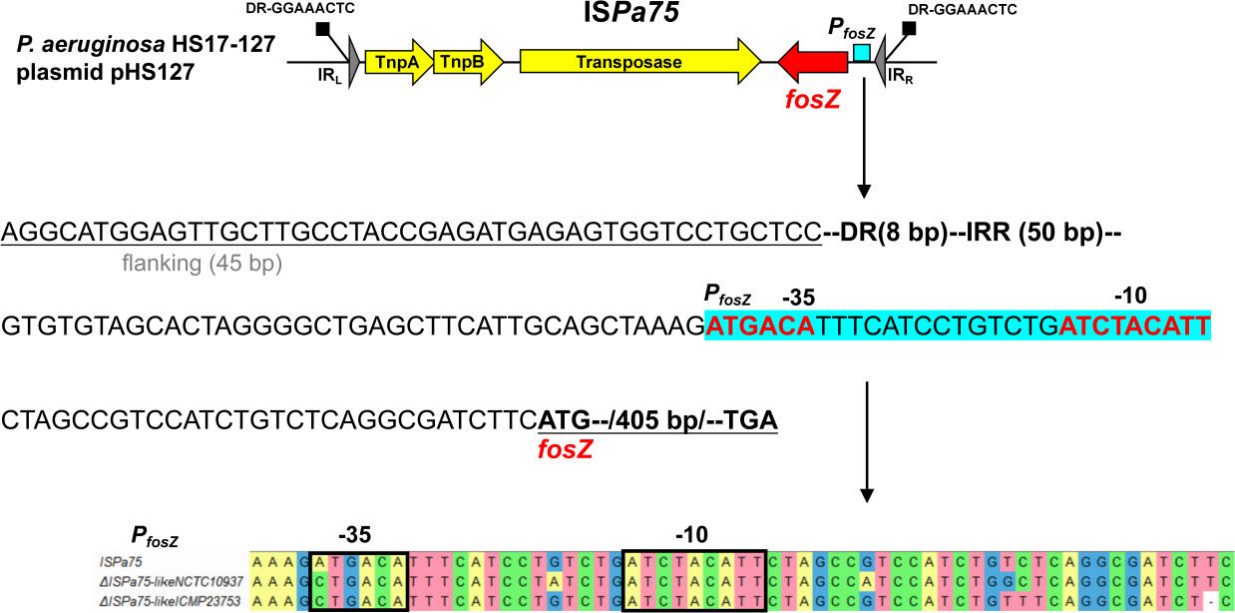
Genetic arrangement of IS*Pa75-fosZ*. This schematic illustrates the location of the BPROM-predicted P*_fosZ_* promoter within IS*Pa75*. The −35 and −10 boxes of the promoter are highlighted in bold and blue. IR_L_, left inverted repeat; IR_R_, right inverted repeat; DR, direct repeat.

The genetic arrangement of IS*Pa75-fosZ* is depicted in Fig. 3. A putative promoter named P*_fosZ_* was identified within IS*Pa75* between the IR_R_ and *fosZ* using BPROM (http://www.softberry.com). Sequence alignment with ΔIS*Pa75*-like_NCTC10937_ and ΔIS*Pa75*-like_ICMP23753_ revealed that P*_fosZ_* is highly conserved (Fig. 3), while the P*_fosZ_* sequence is distinct from the corresponding regions in IS*Psy43* and IS*Pa97*. These findings demonstrate that IS*Pa75* provides the native promoter for *fosZ* and was captured together with it as a composite unit from the original source.

### Characteristics of *fosZ*-bearing plasmids

**Fig 4:**
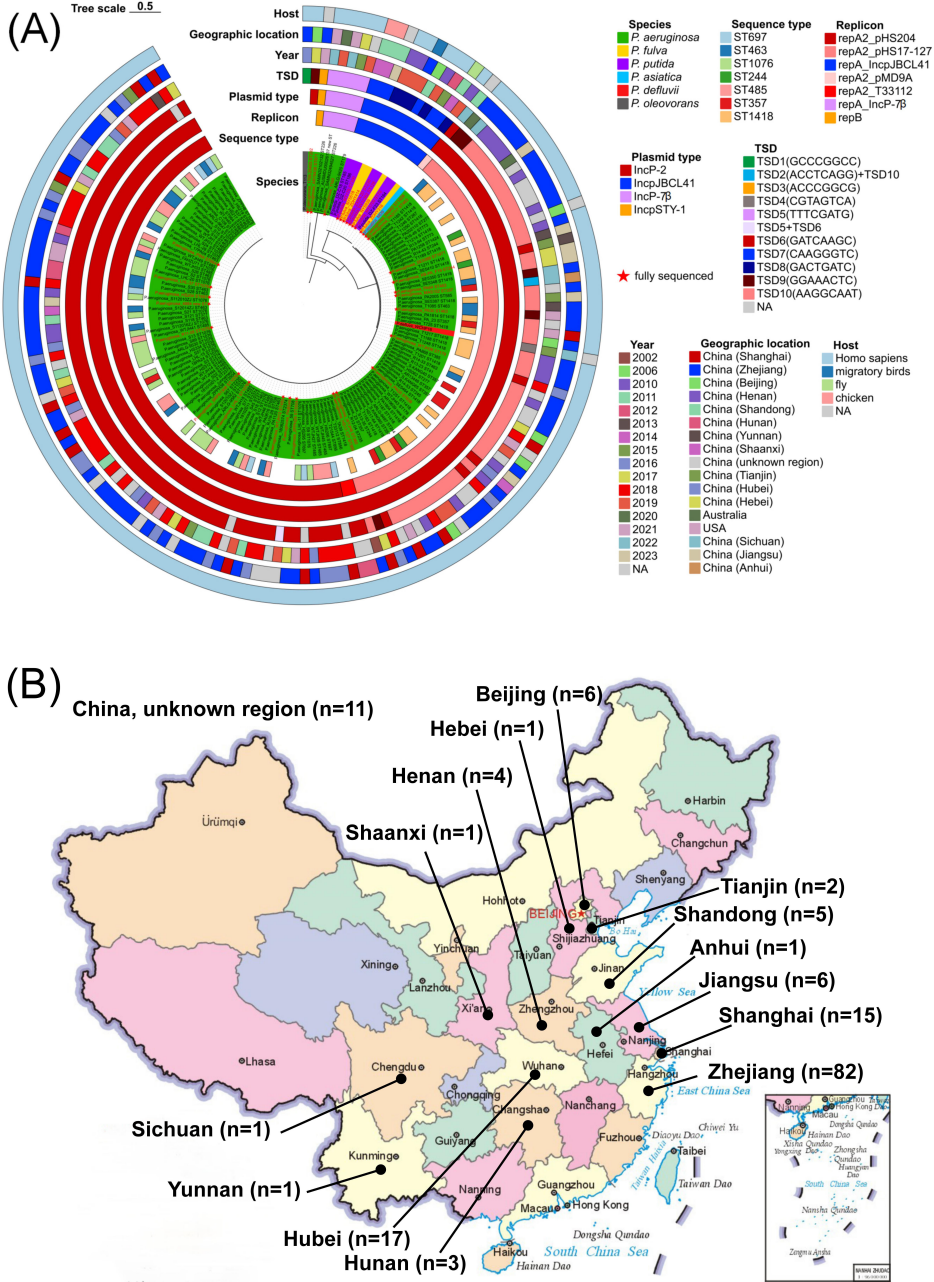
Characteristics of 159 *fosZ*-bearing *Pseudomonas* strains from GenBank worldwide. (**A**) The phylogenetic tree illustrates the replication initiator gene (replicon) of *fosZ*-bearing *Pseudomonas* strains. For *P. oleovorans* T113 and pNY7736-1 (ΔIncP-2), the replicon could not be identified, and these strains are therefore marked solely in the outer ring of the tree. (**B**) Distribution of *fosZ*-bearing *Pseudomonas* strains in China.

We identified ten distinct IS*Pa75* target site duplications (TSDs) among 159 IS*Pa75_fosZ*-bearing *Pseudomonas* strains (Table S4; Fig. 4A). Of these, 34 strains were fully sequenced (Table S6), including 33 plasmids and 2 chromosomes from *P. aeruginosa* strains 59 and MAS152, spanning 4 *Pseudomonas* species and 11 provinces in China between 2011 and 2023 (Fig. 4B; Table S6).

Among the 33 *fosZ*-bearing plasmids, 23 featured typical IncP-2 plasmid modules (17) found in *P. aeruginosa* (*n* = 22) and *P. asiatica* (*n* = 1) and eight were Inc_pJBCL41_ megaplasmids (29) originating from *P. fulva* (*n* = 4) and *P. putida* (*n* = 4) (Table S6). The other two plasmids (p1160-VIM and pAR19438) were classified as IncP-7β (30) and Inc_pSTY-1_ (31) plasmids, respectively. Based on replicon type, backbone, and IS*Pa75* TSDs, these plasmids were categorized into seven groups (A to G) (Table S6). Group A (*n*=6, TSD9: GGAAACTC): IncP-2 plasmids resembling pHS17-127; pNY7736-1 was a disabled IncP-2 (∆IncP-2) plasmid caused by IS*Ppu29*-mediated recombination (Fig. S2). Group B (*n* = 16, TSD6: GATCAAGC): The most prevalent *fosZ*-bearing IncP-2 plasmids across China, carrying various MDR genes (mostly *bla*_KPC-2_) (Fig. S3; Table S6). Moreover, a fused one was observed in the chromosome of *P. aeruginosa* 59 (Fig. S2). Group C (*n*=2, TSD10: AAGGCAAT): IncP-2 plasmids pSE5388-PER and pMAS152 with similar backbones but distinct TSDs with Group A and B (Fig. S4). Group D (*n*=5, TSD8: GACTGATC) and Group E (*n*=3, TSD7: CAAGGGTC) were Inc_pJBCL41_ megaplasmids (Fig. S5) (29, 32), with seven of the eight plasmids co-harboring *bla*_VIM_/*bla*_IMP_ and *tmexCD3-toprJ3* (Table S6). Four Group D plasmids from Henan province showed >99.9% identity, suggesting *P. fulva* clonal spread (Fig. S5; Table S4).

*fosZ* was mostly linked to intact IS*Pa75* elements. Notably, IS*Pa75*s were observed both on the IncP-2 plasmid pMAS152 (at TSD10) and on the chromosome of the same strain, *P. aeruginosa* MAS152, at a new target site (TSD2), indicating mobilization of IS*Pa75* between plasmid and chromosome (Table S6). Disrupted IS*Pa75*s were discovered in pHS204 (Group B) (33), p1160-VIM (Group F), and the chromosome of *P. aeruginosa* strain 59 (Fig. S6). In pHS204, tandem IS*Pa75*s (TSD6) exhibited partial inversion (Fig. S6B). In p1160-VIM, IS*Pa75* (TSD5) was interrupted by an IS*Pa79* variant (Fig. S6C). Interestingly, *P. aeruginosa* strain 59 acquired partial p1160-VIM segments (including ∆IS*Pa75*-TSD5), into which IS*Ppu29* inserted, followed by integration of a Group B plasmid carrying IS*Pa75*-TSD6 (Fig. S6D).

Seven TSDs were identified across the 33 complete sequenced plasmids, associated with four incompatibility types. IncP-2 plasmids carried four TSDs, mainly in *P. aeruginosa* with > 15 STs. TSD9 was carried mainly by IncP-2_*repA2*_pHS17-127 plasmids (5/6) in *P. aeruginosa* especially ST463 strains (3/5), while TSD6 was always carried by IncP-2_*repA2*_pHS204 plasmids (11/16) in *P. aeruginosa* especially ST1076 ones (3/11). There were five nucleotide differences between the *repA2* of pHS17-127 and that of pHS204. TSD7 and TSD8 were associated with Inc_pJBCL41_ plasmids, with TSD8 linked to clonal *P. fulva* strains (Table S4; Fig. 4A).

Similarly, we found IS*Pa75* with the same TSDs among the incompletely sequenced *Pseudomonas* strains carrying plasmids with the same replicons mentioned above. For instance, IS*Pa75*_TSD5s occurred with IncP-7β plasmids (4/4), IS*Pa75*_TSD6s with IncP-2 *repA2* of pHS204 (63/66), and IS*Pa75*_TSD10 with IncP-2 *repA2* of pHS17-127 (46/46). Two novel TSDs were also identified in *P. defluvii* and *P. oleovorans* (Table S4; Fig. 4A).

Among the 159 IS*Pa75_fosZ*-bearing *Pseudomonas* strains, IS*Pa75*_TSD6 was the most prevalent type (82/159), found in at least 21 *P*. *aeruginosa* STs including ST1076 (25/81), ST485 (14/81), and ST463 (12/81) (Fig. 4A; Table S4). IS*Pa75*_TSD10s followed (48/159), recovered in 15 *P*. *aeruginosa* STs including ST1418 (23/48) and ST357 (7/46). *P. aeruginosa* ST463 high-risk clones possessed three TSD types, IS*Pa75*_TSD6s (12/17) associated with IncP-2_*repA2*_pHS204, whereas IS*Pa75*_TSD9s (3/17) and IS*Pa75*_TSD10s (12/17) associated with IncP-2_*repA2*_pHS17-127 (Fig. 4A; Table S4).

Geographically, *fosZ*-bearing *Pseudomonas* strains were almost exclusively from China (98.1%, 156/159), especially Zhejiang province (82/156), Hubei province (17/156), and Shanghai (15/156) (Fig. 4B). In addition to clinical sources, isolates were recovered from environmental settings, sewage, chicken, migratory birds, and flies (Fig. 4A).

### Protein structure analysis of FR-GSTs

**Fig 5:**
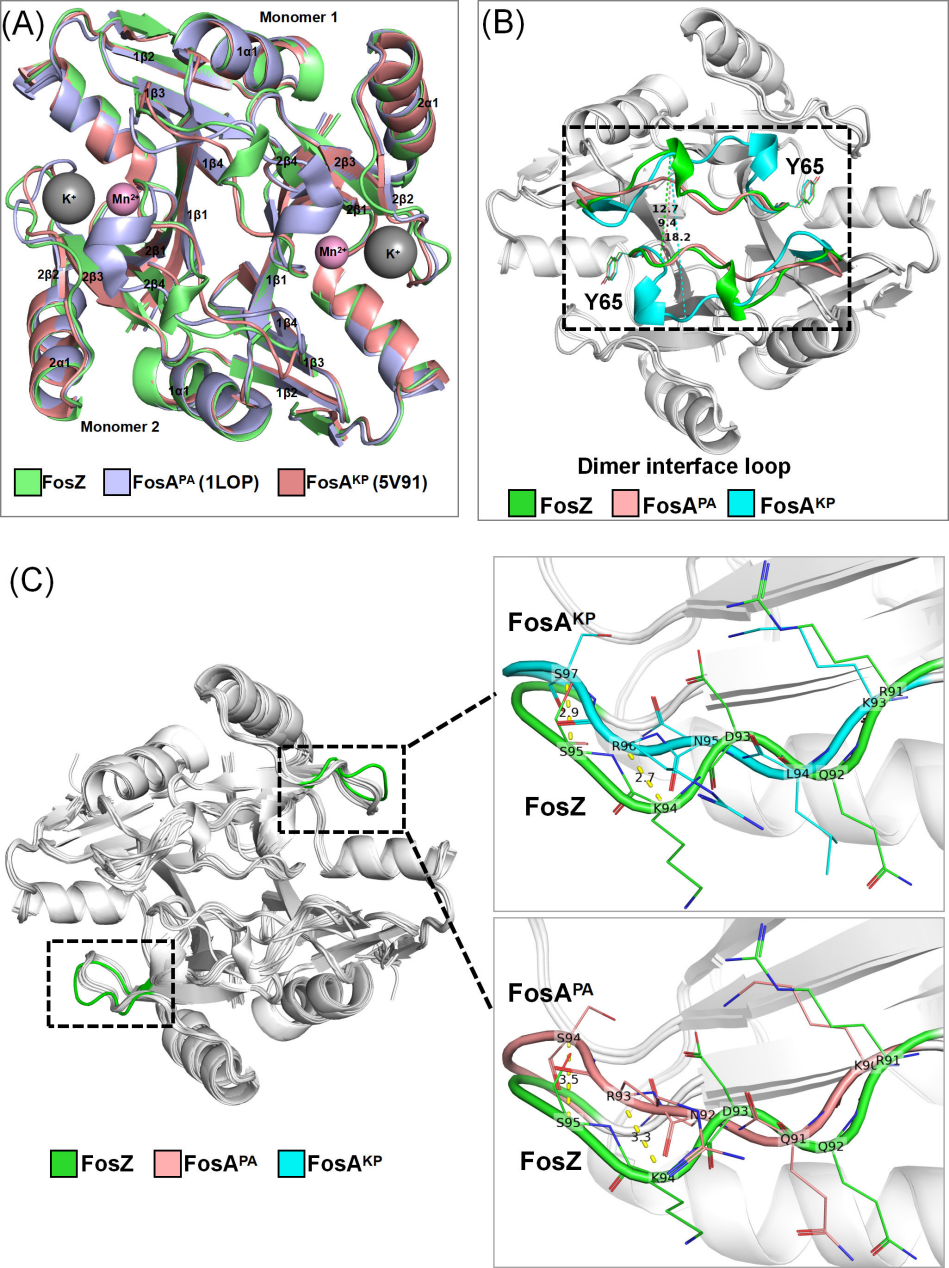
Structural analysis of FosZ. (**A**) An overall alignment of FosZ with previously well-characterized FosA^PA^ (PDB code 1LOP) in *P. aeruginosa* and FosA^KP^ (PDB code 5V91) in *K. pneumoniae*. The superimposition of all three protein structures reveals an RMSD value of less than 0.7 Å between any two of them. Colors listed below each panel correspond to FosA sequences shown in all panels. Mn^2+^ ion and K^+^ ion are shown as colored spheres. (**B**) Comparison of the dimer interface loop in FosZ, FosA,^KP^ and FosA^PA^. The cα RMSD values of loop dimensions in FosZ (12.7 Å, A56 in monomer one to R61 in monomer 2), FosA^PA^ (9.4 Å, G57 in monomer one to A60 in monomer 2), and FosA^KP^ (18.2 Å, V57 in monomer one to P59 in monomer 2) are represented as dashed lines. Y65, which bridges the active site and the dimer interface loop, is labeled as sticks. (**C**) Superimposition of FosZ with predicted FR-GSTs. The black dashed boxes denote expanded K^+^-binding loops in FosZ. The black box represents the K^+^-binding loop residues in FosA^KP^ (R96 to G99), FosA^PA^ (R93 to G96), and FosZ (K94 to G97). K94 in FosZ exhibits a Cα RMSD deviation of 2.7 Å from FosA^KP^ and 3.3 Å from FosA^PA^. S95 in FosZ exhibits a cα RMSD deviation of 2.9 Å from FosA^KP^ and 3.5 Å from FosA^PA^.

We employed ColabFold to predict the protein structures of FosZ (Fig. 5A), FosA-family members (Fig. S7), and other FR-GSTs (Fig. S8). The predicted structures of all dimeric FR-GST proteins exhibited the typical VOC superfamily topology (27), consisting of two βαβββ units that form an incompletely closed β-sheet barrel around the metal ion. The superimposition of FosZ with all predicted FR-GSTs revealed substantial structure conservation, with Cα root mean square deviations (RMSD) values ranging from 0.39 to 0.71 Å.

The length of the FosA dimer interface loop (FosA^KP^ peptide 48-64) impacts enzyme activity, FosA3 and FosA^KP^ with longer and more extended loops conferred greater fosfomycin resistance compared to FosA^PA^ (25). We calculated the Cα RMSD values between residues located at the dimer interface loops to quantify the loop dimensions. All FosA-family members had 17-residue-length dimer interface loops (Fig. 1B), with loop dimensions ranging from 17.5 to 19.4 Å (Fig. S7). To be noted, this loop in FosZ was two amino acid residues shorter than that in FosA^KP^ (with a loop dimension of 12.8 Å) but was longer and more extended than that in FosA^PA^ (Fig. 5B). In the FosL1 structure, the loop was two residues shorter than FosA^KP^ (Fig. 1B), with a loop dimension of 13.7 Å (Fig. S8C), which was similar to FosZ. In the structures of FosC2, FosG, FosF, and FosK, the loops were five amino acid residues shorter than in FosA^KP^ (Fig. 1B), with loops dimensions ranging from 5.3 to 5.4 Å (Fig. S8D through G), and crossed the dimer interface directly like FosA^PA^ (25).

In the binding pocket near the fosfomycin and/or PPF binding site, FosZ had two amino acid substitutions (K93R and N95D, according to the amino acid numbering of FosA^KP^) compared to all FR-GSTs (Fig. 1B). The mobile K^+^-binding loop in FosZ showed deviations when superimposed with all predicted FR-GST structures, exemplified by FosA^KP^ and FosA^PA^ (Fig. 5C). Residue S97 in FosZ was among the four residues involved in binding both fosfomycin and PPF (Fig. 1B) (26).

## DISCUSSION

In recent times, fosfomycin, an antibiotic with a decade-long history, has surged into the spotlight as a potential remedy for combating MDR/XDR bacterial infection. Previous studies (5, 6) have primarily investigated chromosomal mutations as the dominant mechanism of fosfomycin resistance in *P. aeruginosa*. Here, we first characterized the plasmid-borne fosfomycin resistance gene *fosZ* encoding glutathione S-transferase FosZ, which conferred fosfomycin resistance in *P. aeruginosa*. The *fosZ* gene was captured by translocatable genetic element IS*Pa75*, which may facilitate the dissemination of fosfomycin resistance among different *Pseudomonas* species, predominantly through IS*Pa75*-medicated transposition within IncP-2 plasmids among *P. aeruginosa*, particularly in MBL-positive isolates. An IncP-2 plasmid sublineage has been linked to the dissemination of *bla*_IMP-45_ among CRPA in the intensive care unit (ICU) of a hospital in Shanghai, China (17). IncP-2 plasmid has accumulated multiple resistance genes during its evolution, including those conferring resistance to aminoglycosides, antipseudomonal carbapenems, cefiderocol (*bla*_PER-1_), ceftolozane-tazobactam (*bla*_KPC-33_), and fosfomycin, facilitating cross-species transfer of resistance genes. The *fosZ*-bearing Inc_pJBCL41_ megaplasmid pZXPA-20-602k from *P. putida* of migratory bird origin has been reported as a convergence of *bla*_VIM-2_ and *tmexCD1-toprJ1* (32). We observed the spread of Inc_pJBCL41_ megaplasmid from *P. fulva*, which co-carried *bla*_VIM-24_, *tmexCD3-toprJ3*, and *fosZ* in a hospital in Henan, China, in 2019. These MDR megaplasmids may serve as reservoirs for resistance genes against carbapenems, tigecycline, and fosfomycin, presenting significant obstacles for clinical treatment and infection control.

Fosfomycin combined with new antimicrobials is gaining attention for treating MDR-PA infections (34, 35). The activity of such regimens in *P. aeruginosa* infections remains debated and has been explored in several *in vitro* studies. The ceftazidime/avibactam and fosfomycin combination significantly reduced *P. aeruginosa* colony-forming units, surpassing individual drugs and offering a practical alternative for MBL-negative isolates (3). The ceftolozane/tazobactam and fosfomycin combination displayed synergy in time-kill analyzes against MBL-positive isolates (4). CLSI lacks fosfomycin breakpoints specific for *P. aeruginosa*. European Committee on Antimicrobial Susceptibility Testing (EUCAST) mentions that (36), for wild-type *P. aeruginosa* isolates (with an epidemiological cutoff value of MIC 128 mg/L), fosfomycin has been employed in combination therapy. Our findings provide further evidence of transferable sources of fosfomycin resistance among *Pseudomonas* species. This may limit its clinical applicability in combination therapy for MDR-PA infections.

PPF (Foscarnet) is clinically used mainly for treating viral infections. *In vitro* studies suggest that at therapeutic plasma concentrations, PPF can enhance fosfomycin’s activity against some bacteria that produce GST (Table S1), potentially reversing fosfomycin resistance in GST-producing Gram-negative bacteria. This synergy requires further clinical validation but highlights a novel combinatorial strategy against resistant pathogens (15, 16). Our findings suggest that FosZ cannot be inhibited by PPF, which may be attributed to the structural differences between FosZ and other FR-GSTs like FosA^KP^ and FosA^PA^. FosZ exhibits an expanded K^+^-binding loop pocket, leading to deviation of residue S97, which participates in binding both the inhibitor and substrate (26). This structural alteration may reduce the competitive inhibition efficacy of PPF against fosfomycin. However, this remains a structural speculation and necessitates further biochemical evidence to support it.

Our BLAST analysis identified FosZ-like proteins in the chromosomes of many *Pseudomonas* species, suggesting that *fosZ* likely originated from *Pseudomonas* species. IS*66* family elements (e.g., unknown IS in *Pseudomonas* spp. SWI36, and IS*Psko1*) likely captured a *fosZ*-like sequence, leading to the formation of IS*Pa75*.

Some FosA variants such as FosA1, FosA2, and FosA11 exhibit amino acid homology below 80% or 70% with other FosA-family members but share similar protein structures, notably in the 17-residue-length dimer interface loops. The dimer interface loop serves as a key structural feature of FR-GSTs, distinguishing FosA-family members from other FR-GSTs, such as FosZ, which has a 15-residue-length loop.

In our recent research, we identified eight IncP-2 plasmids from CRPA isolates carrying *bla*_PER-1_ and IS*Pa75-fosZ* (33). The *fosZ* gene, trapped in IS*Pa75*, is now prevalent across both southern and northern China, particularly in Zhejiang province, Hubei province, and Shanghai, over the past 14 years (2011 to 2024). IS*Pa75* has been transferred among different plasmid incompatible groups, including IncP-2, Inc_pJBCL41_, IncP-7β, and Inc_pSTY-1_, and between plasmid and chromosome within the same isolate. The presence of multiple IS*Pa75* target sites across different plasmids, coupled with its relocation to various positions within plasmids of the same incompatibility type, demonstrates IS*Pa75*’s high transferability. This mobility facilitates the dissemination of resistance genes among diverse species. Furthermore, this may reflect the evolutionary pressures faced by *Pseudomonas* species, which enables them to adapt quickly to changing environments and enhances their survival in the presence of antimicrobial agents. Group B plasmids, the most common *fosZ*-bearing IncP-2 types, have accumulated various MDR genes and have been captured by high-risk ST463 clones.

In summary, the emergence of the plasmid-borne fosfomycin resistance determinant *fosZ* poses critical concerns in antimicrobial stewardship, particularly given fosfomycin’s indispensable role as a combinatorial agent against MDR-PA isolates, specifically MBL-producing ones with extremely restricted treatment options.

## Data Availability

The full sequences of *fosZ* have been submitted to GenBank and assigned accession number OR827686.

## SUPPLEMENTAL MATERIAL

Supplemental material aac.00750-25-s0001.pdf Tables S1 to S3, S5 and S6; Fig. S1 to S8.

Table S4 aac.00750-25-s0002.xlsx Characteristics of 159 fosZ-bearing *Pseudomonas* strains (160 sequences) from GenBank worldwide.

ASM does not own the copyrights to Supplemental Material that may be linked to, or accessed through, an article. The authors have granted ASM a non-exclusive, world-wide license to publish the Supplemental Material files. Please contact the corresponding author directly for reuse.
