# Supplementary material for: fosZ, a novel plasmid-borne fosfomycin resistance gene in Pseudomonas species, especially carbapenem-resistant Pseudomonas aeruginosa isolates
Source: Antimicrob Agents Chemother. 2026 Jan 14;70(2):e00750-25. doi: 10.1128/aac.00750-25 (PMC12888890; doi:10.1128/aac.00750-25)
Supplement: Supplemental material — Tables S1 to S3, S5 and S6; Fig. S1 to S8. [file aac.00750-25-s0001.pdf]

fosZ, a novel plasmid-mediated fosfomycin resistance gene in *Pseudomonas* species especially carbapenem-resistant *Pseudomonas aeruginosa* isolates

Table S1 Characteristics of the known fosfomycin-modifying enzymes

Enzyme	GenBank Protein ID	Nature of the enzyme	PPF inhibition test	Bacterium	Remark (likely species of origin)	Reference
FosA1	AAA98399	Glutathione-S-transferase	ND	*Serratia marcescens*	Acquired/plasmid, *Enterobacter cloacae*	(1)
FosA2	ACC85616	Glutathione-S-transferase	ND	*Enterobacter cloacae*	*Enterobacter cloacae*	(2)
FosA3	ALA99215	Glutathione-S-transferase	Inhibited	*Escherichia coli*	Acquired/plasmid, *Kluyvera georgiana*	(3)
FosA4	BAP18892	Glutathione-S-transferase	Inhibited	*Escherichia coli*	Acquired/plasmid, *Kluyvera georgiana*	(4)
FosA5	AJE60855	Glutathione-S-transferase	ND	*Escherichia coli*	Acquired/plasmid, *Klebsiella pneumoniae*	(5)
FosA6	AMQ12811	Glutathione-S-transferase	Inhibited	*Escherichia coli*	Acquired/plasmid, *Klebsiella pneumoniae*	(6)
FosA7	KKE03230	Glutathione-S-transferase	Inhibited	*Salmonella enterica* serovar Heidelberg	*Salmonella enterica* serovar Heidelberg	(7)
FosA8	QEI22965	Glutathione-S-transferase	Inhibited	*Escherichia coli*	Acquired/plasmid, *Leclercia adecarboxylata*	(8)
FosA9	WP_114473955	Glutathione-S-transferase	ND	*Escherichia coli*	Acquired/plasmid, *Klebsiella variicola*	(9)

FosA10	QIS31064	Glutathione-S-transferase	ND	*Escherichia coli*	Acquired/plasmid, *Klebsiella pneumoniae*	(10)
FosA11	QZL11398	Glutathione-S-transferase	Inhibited	*Providencia rettgeri*	Acquired/plasmid	(11)
FosC2	BAJ10053	Glutathione-S-transferase	Inhibited	*Escherichia coli*	Acquired/plasmid	(3)
FosF	AAP50248	Glutathione-S-transferase	ND	*Pseudomonas aeruginosa*	Acquired/Integron	(12)
FosG	AAZ14834	Glutathione-S-transferase	ND	*Achromobacter denitrificans*	Acquired/plasmid	Unpublished，reviewed in (13) and (14)
FosK	BAO79518	Glutathione-S-transferase	ND	*Acinetobacter soli*	Acquired/Integron	(15)
FosL1	QHR93773	Glutathione-S-transferase	Inhibited	*Escherichia coli*	Acquired/plasmid	(13)
FosZ	WXA37068	Glutathione-S-transferase	Not inhibited	*Pseudomonas aeruginosa*	Acquired/plasmid	This study
FosB	CAA38136	Bacillithiol transferase	/	*Staphylococcus epidermidis*	Acquired and inherent	(16)
FosD	AHB87392	Bacillithiol transferase	/	*Staphylococcus rostri*	/	(13, 14)
FosE	BAO48025	Bacillithiol transferase	/	*Pseudomonas aeruginosa*	/	(13, 14)
FosH	ABD22979	Bacillithiol transferase	/	*Pseudomonas aeruginosa*	/	(13, 14)
FosI	AFJ38137	Bacillithiol transferase	/	*Mycobacterium abscessus*	Acquired/Integron	(17)

FosM	DAC85639	Bacillithiol transferase	/	*Escherichia coli*	/	(18)
FosY	QTE33800	Bacillithiol transferase	/	*Staphylococcus aureus*	Acquired/plasmid	(19)
FosX	WP_003726635	Mn^{2+} dependent hydrolase	/	*Listeria monocytogenes*	/	(20)

Table S2. Bacterial isolates, plasmids and primers used in this study

Isolates and plasmids	Characteristics	Source
Isolates		
P. aeruginosa HS17-127	Clinical isolate	Laboratory stock, Ref (21)
P. aeruginosa HS355	Clinical isolate	Laboratory stock
E. cloacae 19-4074	Clinical isolate	Laboratory stock
TcHS17-127	*P. aeruginosa* PAO1 transconjugant harbouring plasmid pHS17-127 from isolate HS17-127.	Laboratory stock, Ref (21)
P. aeruginosa PAO1	Cloning host	Laboratory stock
Plasmids		
pUCP19	*Escherichia-Pseudomonas* shuttle vector; ampicillin and carbenicillin	Shanghai Bioresource Collection Center, China
pUCP19-*fosZ*	pUCP19 carrying the full ORF of *fosZ*	This study
pUCP19-pro-*fosZ*	pUCP19 carrying the full ORF of *fosZ* and its native promoter	This study
**Primers**	**Sequences (5'-3')***	**Purpose**
fosZ-HindIII-F	CCCAAGCTTATGCTGACTGGCTTCAATCA	Cloning of *fosZ* into vector pUCP19
fosZ-BamHI-R	CGCGGATCCTCAGTCGAAAAACTCCATCTCG	
pro-*fosZ*-HindIII-F	CCCAAGCTTGGCATGGAGTTGCTTGCCTA	Cloning of pro-*fosZ* into vector pUCP19

*Underlined sequences mark restriction cutting sites for HindIII and BamHI respectively.

Table S3 FosZ-related proteins with identities more than 70% in Nonredundant Protein Database using BLASTP (Oct 9, 2024)

No.	Accession	Accession Length	Query Coverage	Percent Identity	Scientific Name	Description
1	WP_065760318.1	136	100%	100.00%	*Pseudomonas*	VOC family protein [Pseudomonas]
2	WP_238216096.1 /GHS83130.1	136	100%	89.71%	*Pseudomonas sp*. PAGU 2196	VOC family protein
3	WP_133752033.1	136	100%	87.50%	*Pseudomonas sp*. LP_7_YM	VOC family protein
4	WP_248919709.1	136	100%	78.68%	*Pseudomonas entomophila*	fosfomycin resistance glutathione transferase
5	WP_213660662.1	136	100%	78.68%	*Pseudomonas entomophila*	fosfomycin resistance glutathione transferase
6	WP_240063401.1	136	100%	77.94%	*Pseudomonas entomophila*	fosfomycin resistance glutathione transferase
7	WP_252088756.1	136	100%	77.94%	*Pseudomonas* sp. MWU13-3659	fosfomycin resistance glutathione transferase
8	WP_011533467.1	136	100%	77.21%	*Pseudomonas entomophila*	fosfomycin resistance glutathione transferase
9	WP_277459859.1	136	100%	76.47%	*Pseudomonas entomophila*	fosfomycin resistance glutathione transferase
10	WP_186657843.1	136	100%	75.00%	*Pseudomonas xantholysinigenes*	fosfomycin resistance glutathione transferase
11	WP_134693502.1	136	100%	72.79%	Pseudomonas sp. RIT623	fosfomycin resistance glutathione transferase
12	WP_341958568.1	136	100%	76.47%	Pseudomonas sp. RC10	fosfomycin resistance glutathione transferase
13	WP_217848044.1	136	100%	72.79%	*Pseudomonas muyukensis*	fosfomycin resistance glutathione transferase
14	WP_253093754.1	136	100%	72.06%	*unclassified Pseudomonas*	fosfomycin resistance glutathione transferase
15	WP_217865943.1	136	100%	76.47%	*Pseudomonas maumuensis*	fosfomycin resistance glutathione transferase
16	WP_182324244.1	136	99%	73.33%	*Pseudomonas mosselii*	fosfomycin resistance glutathione transferase
17	WP_186735237.1	136	100%	72.79%	*Pseudomonas peradeniyensis*	fosfomycin resistance glutathione transferase
18	WP_062363135.1	136	99%	72.59%	*Pseudomonas mosselii*	fosfomycin resistance glutathione transferase
19	WP_203646610.1	136	100%	73.53%	unclassified *Pseudomonas*	fosfomycin resistance glutathione transferase
20	WP_236180884.1	136	99%	73.33%	*Pseudomonas mosselii*	fosfomycin resistance glutathione transferase
21	WP_234561149.1	136	100%	72.79%	Pseudomonas sp. AA27	fosfomycin resistance glutathione transferase
22	WP_088852520.1	136	98%	73.88%	*Pseudomonas*	fosfomycin resistance glutathione transferase

23	WP_094009989.1	136	100%	73.53%	*Pseudomonas soli*	fosfomycin resistance glutathione transferase
24	WP_254463748.1	136	100%	72.79%	unclassified Pseudomonas	fosfomycin resistance glutathione transferase
25	WP_280081861.1	136	100%	72.79%	*Pseudomonas mosselii*	fosfomycin resistance glutathione transferase
26	WP_262951461.1	136	100%	72.79%	*Pseudomonas peradeniyensis*	fosfomycin resistance glutathione transferase
27	AIN58681.1	136	100%	72.79%	*Pseudomonas soli*	glutathione transferase
28	WP_236248745.1	136	98%	73.13%	*Pseudomonas mosselii*	fosfomycin resistance glutathione transferase
29	WP_111532809.1	136	100%	72.79%	*Pseudomonas*	fosfomycin resistance glutathione transferase
30	WP_349738970.1	136	99%	71.85%	*Pseudomonas mosselii*	fosfomycin resistance glutathione transferase
31	WP_186713401.1	136	100%	72.06%	Pseudomonas sp. BW16M2	fosfomycin resistance glutathione transferase
32	WP_028688971.1	136	99%	72.59%	*Pseudomonas mosselii*	fosfomycin resistance glutathione transferase
33	WP_207852186.1	136	99%	72.59%	*Pseudomonas mosselii*	fosfomycin resistance glutathione transferase
34	WP_277504034.1	136	100%	72.06%	*Pseudomonas hunanensis*	fosfomycin resistance glutathione transferase
35	WP_330126075.1	136	100%	72.79%	*Pseudomonas soli*	fosfomycin resistance glutathione transferase
36	WP_110604619.1	136	100%	72.06%	*Pseudomonas soli*	fosfomycin resistance glutathione transferase
37	WP_367855982.1	136	100%	72.06%	*Pseudomonas sp.* TE50-2	fosfomycin resistance glutathione transferase
38	WP_267302807.1	136	100%	72.06%	*Pseudomonas sp.* BJa3	fosfomycin resistance glutathione transferase
39	WP_046855502.1	136	100%	72.06%	*Pseudomonas sp.* CCOS 191	fosfomycin resistance glutathione transferase
40	WP_330102474.1	136	100%	72.06%	*Pseudomonas carassii*	fosfomycin resistance glutathione transferase
41	WP_198755061.1	136	100%	71.32%	*Pseudomonas sp.* CCOS 191	fosfomycin resistance glutathione transferase
42	WP_110738884.1	136	100%	71.32%	*Pseudomonas mosselii*	fosfomycin resistance glutathione transferase
43	WP_104443796.1	136	100%	72.79%	*Pseudomonas sp.* PONIH3	fosfomycin resistance glutathione transferase
44	WP_110701270.1	136	100%	72.06%	*Pseudomonas soli*	fosfomycin resistance glutathione transferase
45	WP_166891913.1	136	100%	71.32%	*Pantoea sp.* Cy-639（*Enterobacterales; Erwiniaceae; Pantoea*）	fosfomycin resistance glutathione transferase
46	WP_279886118.1	134	100%	70.59%	unclassified *Pseudomonas*	fosfomycin resistance glutathione transferase

Table S5. IS*66* family members in *Pseudomonas* species in ISfinder database

No.	Insertion sequence (IS) name	Remark	Species	IS sequence length (bp)	IR length (bp)	DR length (bp)	Passenger gene annotation	Accession
1	IS*Pa75*	*t*IS*66*	*Pseudomonas aeruginosa*	2996	23/33	8	FosZ, VOC family protein	MF144194
2	IS*Psp18*	*t*IS*66*	*Pseudomonas* sp.	2946	18/21	8	GNAT family N-acetyltransferase	ND*
3	IS*Pa128*	*t*IS*66*	*Pseudomonas aeruginosa*	2876	23/28	8	GNAT family N-acetyltransferase	CP054623.1
4	IS*Pa120*	*t*IS*66*	*Pseudomonas aeruginosa*	2880	20/24	8	Hypothetical protein	CP047592.1
5	IS*Pre3*	*t*IS*66*	*Pseudomonas resinovorans*	2957	17/24	8	Hypothetical protein	NC_004444
6	IS*Ppu30*	*t*IS*66*	*Pseudomonas putida*	3000	20/23	8	Hydrolase	CP016215
7	IS*Psy43*	*t*IS*66*	*Pseudomonas syringae*	4875	17	8	Methyl-accepting chemotaxis protein	CP012179
8	IS*Psko1*	IS*66*	*Pseudomonas koreensis*	2383	26/27	8	/	ND*
9	IS*Ppu14*	IS*66*	*Pseudomonas putida*	2383	27/33	8	/	NC_002947
10	IS*Pa97*	IS*66*	*Pseudomonas aeruginosa*	2361	23/29	8	/	EU595745
11	IS*Pa160*	IS*66*	*Pseudomonas aeruginosa*	2553	23/40	ND*	/	ND*
12	IS*Pa122*	IS*66*	*Pseudomonas aeruginosa*	2358	17/24	ND*	/	ND*
13	IS*Pa140*	IS*66*	*Pseudomonas aeruginosa*	2384	21/24	8	/	MN894887
14	IS*Ppu19*	IS*66*	*Pseudomonas putida*	2377	23	8	/	AB238971
15	IS*684*	IS*66*	*Pseudomonas syringae*	2040	21/30	2	/	AF232005
16	IS*Pa109*	IS*66*	*Pseudomonas aeruginosa*	2051	22/25	8	/	ND*
17	IS*Pa134*	IS*66*	*Pseudomonas aeruginosa*	2065	25/31	8	/	ND*
18	IS*Pa30*	IS*66*	*Pseudomonas aeruginosa*	2436	20/24	8	/	DQ315788
19	IS*Pa82*	IS*66*	*Pseudomonas aeruginosa*	2017	19/25	ND*	/	ND*
20	IS*Ppu13*	IS*66*	*Pseudomonas putida*	2370	19/22	8	/	NC_002947
21	IS*Ppu15*	IS*66*	*Pseudomonas putida*	2041	22/28	8	/	NC_002947
22	IS*Psas1*	IS*66*	*Pseudomonas asiatica*	2668	73/96	8	/	CP128508

23	IS*Psp17*	IS*66*	*Pseudomonas* sp.	2340	24/25	8	/	ND*
24	IS*Psy34*	IS*66*	*Pseudomonas syringae*	2041	22/32	8	/	ND*
25	IS*Psy5*	IS*66*	*Pseudomonas syringae*	2059	21/28	8	/	ND*

*ND, not determined.

ISfinder: https://www-is.biotoul.fr.

Table S6. Characteristics of 35 completely sequenced *fosZ*-bearing plasmids and chromosomes of *Pseudomonas* strains from GenBank.

Group	No.	Host bacterium	Sequence type*	Plasmid name	Accession number	Plasmid type	Plasmid size (bp)	Isolation time and location in China (reference)	IS*Pa75* target sites (TSDs)	Antimicrobial resistance genes								
										Aminoglycosides	β-lactams	Fluoroquinolones	Macrolides	Chloramphenicol	Rifampicin	Sulphonamides	Trimethoprim	Tetracyclines
	1	*P. aeruginosa* HS17-127	ST369	pHS17-127	CP061377	IncP-2	486,963	2017, Shanghai (22)	TSD9 (GGAAACTC)	*aac(6')-Ib3, aph(3')Ia, armA*	bla_{OXA-1}, bla_{IMP-45}, bla_{PER-1}, bla_{AFM-1}	*qnrVC6*	*msr(E), mph(E)*	*catB3*	/	*sul1*	/	*tet(C)*
	2	*P. aeruginosa* PA30	ST463	pPA30_1	CP104871	IncP-2	453,250	2021, Zhejiang (23)	TSD9 (GGAAACTC)	*aac(6')-Ib3,aph(3')-Ia, armA*	bla_{OXA-1}, bla_{IMP-45}, bla_{PER-1}, bla_{AFM-1}	*qnrVC6*	*msr(E), mph(E)*	*catB3*	/	*sul1*	/	*tet(C)*
Group A (n=6)	3	*P. aeruginosa* PA3117	ST463	pPA3117_1	CP159842	IncP-2	449,377	2022, Zhejiang	TSD9 (GGAAACTC)	*aac(6')-Ib3, aph(3')-Ia armA*	bla_{OXA-1}, bla_{IMP-45}, bla_{PER-1}, bla_{AFM-1}	*qnrVC6*	*msr(E), mph(E)*	*catB3*	/	*sul1*	/	*tet(C)*
	4	*P. aeruginosa* PA942	ST1428	pPA942-IMP45	CP129201	IncP-2	506,353	2020, Zhejiang (24)	TSD9 (GGAAACTC)	*aac(6')-Ib3,aph(3')-Ia, armA*	bla_{OXA-1}, bla_{IMP-45}, bla_{PER-1}, bla_{AFM-1}	/	*msr(E), mph(E)*	*catB3*	/	*sul1*	/	*tet(C)*
	5	*P. aeruginosa* PA64	ST463	pPA64-1	CP159838	IncP-2	426,741	2022, Zhejiang	TSD9 (GGAAACTC)	*aac(6')-Ib3, aph(3')-Ia armA*	bla_{OXA-1}, bla_{IMP-45}	/	*msr(E), mph(E)*	*catB3*	/	*sul1*	/	*tet(C)*
	6	*P. aeruginosa* 1709-25403	ST292	pNY7736-1	CP131615	ΔIncP-2 (disabled)	413,844	2017, Beijing	TSD9 (GGAAACTC)	*aac(6')-Ib3, aph(3')-Ia armA*	bla_{OXA-1}, bla_{IMP-45}, bla_{PER-1}, bla_{AFM-1}	*qnrVC6*	*msr(E), mph(E)*	*catB3*	/	*sul1*	/	*tet(C)*
	7	*P. aeruginosa* PAB546	NA	pNK546-KPC	MN433457	IncP-2	475,027	Tianjin (time unknown)	TSD6 (GATCAAGC)	*ant(2'')-Ia*	bla_{KPC-2}	/	/	*cmlA1*	/	*sul1*	/	/
	8	*P. aeruginosa* P9W	ST664	plasmid unnamed1	CP081203	IncP-2	475,028	2018, Tianjin (26)	TSD6 (GATCAAGC)	*ant(2'')-Ia*	bla_{KPC-2}	/	/	*cmlA1*	/	*sul1*	/	/
	9	*P. aeruginosa* WTJH12	ST485	pWTJH12-KPC	CP064404	IncP-2	396,963	2018, Hubei	TSD6 (GATCAAGC)	/	bla_{KPC-2}	/	/	/	/	/	/	/
	10	*P. aeruginosa* ZPPH29	ST3504	pZPPH29-KPC	CP077978	IncP-2	397,554	2017, Zhejiang	TSD6 (GATCAAGC)	/	bla_{KPC-2}	/	/	/	/	/	/	/
	11	*P. aeruginosa* R11-08	ST1076	pR11-08	CP137894	IncP-2	395,022	Zhejiang (time unknown)	TSD6 (GATCAAGC)	/	bla_{KPC-2}	/	/	/	/	/	/	/
Group B (n=15)	12	*P. aeruginosa* 4884	new, ST-315 (3 mis)	pL4884	CP160408	IncP-2	394,108	2023, Zhejiang	TSD6 (GATCAAGC)	/	bla_{KPC-2}	/	/	/	/	/	/	/
	13	*P. aeruginosa* SRPA1308	ST1076	pSRPA1308	CP158571	IncP-2	394,374	Zhejiang (time unknown)	TSD6 (GATCAAGC)	/	bla_{KPC-33}	/	/	/	/	/	/	/
	14	*P. aeruginosa*	NA	plasmid unnamed 2	OL468012	IncP-2	394,374	unknown	TSD6 (GATCAAGC)	/	bla_{KPC-33}	/	/	/	/	/	/	/
	15	*P. aeruginosa* LHL	ST1076	pLHL1-KPC-3	CP099961	IncP-2	394,987	2021, Henan	TSD6 (GATCAAGC)	/	bla_{KPC-33}	/	/	/	/	/	/	/
	16	*P. aeruginosa* PAG5	ST639	pPAG5	CP045003	IncP-2	513,322	2016, Shaanxi (25)	TSD6 (GATCAAGC)	*aac(6')-Ib3, aph(3')-Ia,armA*	bla_{OXA-1},bla_{IMP-45}	*qnrVC1*	*msr(E), mph(E)*	*catB3*	*ARR-2*	*sul1*	*dfrA22*	/
	17	*P. aeruginosa* KB-PA_F19	ST244	pKB-PA_F19-4	CP086014	IncP-2	412,187	2015, Yunnan	TSD6 (GATCAAGC)	*aac(6')-Ib3, aph(3')-Ia,armA*	bla_{OXA-1}, bla_{IMP-45}	*qnrVC6*	*msr(E), mph(E)*	*catB3*	/	*sul1*	/	*tet(C), tmexCD3-TOpr*

																		J3
	18	*P. aeruginosa* WTJH6	ST360	pWTJH6	CP104587	IncP-2	426,499	2018, Hubei	TSD6 (GATCAAGC)	*aac(6')-Ib3,aph(6)-Id ,aph(3'')-Ib,aadA1*	$bla_{OXA-101}$,bla_{VIM-24} , bla_{VIM-36}	/	/	/	/	*sul1*	/	/
	19	*P. aeruginosa* WTJH36	ST179	pWTJH36	CP104591	IncP-2	462,066	2018, Hubei	TSD6 (GATCAAGC)	*aac(6')-Ib3,aph(6)-Id ,aph(3'')-Ib,aadA1*	$bla_{OXA-101}$,bla_{VIM-24} , bla_{VIM-36}	/	/	/	/	*sul1*	/	/
	20	*P. aeruginosa* HS204	ST697	pHS204	CP110191	IncP-2	416,612	2018, Shanghai (27)	TSD6 (GATCAAGC)	*aac(6')-Ib3*	bla_{PER-1}	*qnrVC6*	/	/	/	*sul1*	/	/
	21	*P. asiatica* MD9	NA	pMD9A	CP101701	IncP-2	455,169	2022, Shandong	TSD6 (GATCAAGC)	*aac(3)-IId,aac(6')-IIa,aac(6')-II, aph(6)-Id,aph(3'')-Ib ,aph(3')-VI, aadA24*	$bla_{OXA-246}$, bla_{AFM-1}	*qnrVC1*	*msr(E), mph(E), mph(A)*	*cmlA1*	*ARR-2, ARR-3*	*sul1*	*dfrA22 dfrA27*	/
	22	*P. aeruginosa* strain 59	ST708	PA59	CP123953	IncP-2 (embedded)	439, 094	2019, Shanghai	TSD6 (GATCAAGC) and TSD5(TTTCGATG)	*aac(3)-IId, aadA1, rmtB*	bla_{TEM-1B}	*qnrVC6*	*mph(A), ere(A)*	*catB3,*	/	*sul1*	/	/
			ST708	/	CP123953	Chromosome	/	2019, Shanghai		/	/	/	/	/	/	/	/	/
Group C (n=2)	23	*P. aeruginosa* SE5388	ST1971	pSE5388-PER	MT598646	IncP-2	429,121	Beijing (time unknown)	TSD10 (AAGGCAAT)	*aac(3)-IId,aac(6')-IIa, aph(6)-Id,aph(3'')-Ib , aadA13,rmtB*	bla_{TEM-1B}, $bla_{OXA-246}$, bla_{PER-1}	/	/	/	/	*sul1*	/	*tmexCD3-TOprJ3*
	24	*P.aeruginosa* MAS152	ST357	pMAS152	CP139037.1	IncP-2	422,428	2022, Anhui	TSD10 (AAGGCAAT)	*aac(3)-IId,aac(6')-IIa, aadA13, rmtB*	bla_{TEM-1B}, $bla_{OXA-246}$, bla_{PER-1}	/	/	/	/	*sul1*	/	*tmexCD3-TOprJ3*
	25	*P.aeruginosa* MAS152	ST357	/	/	Chromosome	/	2022, Anhui	TSD2 (ACCTCAGG)	/	/	/	/	/	/	/	/	/
Group D (n=5)	26	*P. fulva* ZDHY316	NA	pVIM-24-ZDHY316	CP064945	Inc$_{pJBCL41}$	585,396	2019, Henan, bile specimen of liver transplant patient	TSD8 (GACTGATC)	*aac(6')-IIc,aac(6')-IIa,aac(6')-Ib3,aph(6)-Id,aph(3'')-Ib,aph(3')-VI,aadA1*	bla_{OXA-10},bla_{VIM-24}	*qnrVC6*	*msr(E), mph(E)*	/	*ARR-3*	*sul1*	/	*tmexCD3-TOprJ1*
	27	*P. fulva* ZDHY414	NA	pVIM-24-ZDHY414	CP064948	Inc$_{pJBCL41}$	589,460	2019, Henan, pleural fluid in respiratory ICU	TSD8 (GACTGATC)	*aac(6')-IIc,aac(6')-IIa,aac(6')-Ib3,aph(6)-Id,aph(3'')-Ib,aph(3')-VI,aadA1*	bla_{OXA-10},bla_{VIM-24}	*qnrVC6*	*msr(E), mph(E)*	/	*ARR-3*	*sul1*	/	*tmexCD1-TOprJ1*
	28	*P.fulva* NY5518	NA	pNY5518-VIM	CP162503	Inc$_{pJBCL41}$	581,766	2019, Henan, blood	TSD8 (GACTGATC)	*aac(6')-IIc,aac(6')-IIa,aac(6')-Ib3,aph(6)-Id,aph(3'')-Ib,aph(3')-VI,aadA1*	bla_{OXA-10},bla_{VIM-24}	*qnrVC6*	*msr(E), mph(E)*	/	*ARR-3*	*sul1*	/	*tmexCD1-TOprJ1*
	29	*P. fulva* NY5939	NA	pNY5939-OXA	CP161880	Inc$_{pJBCL41}$	577,808	2019, Henan, bile	TSD8 (GACTGATC)	*aac(6')-IIc,aac(6')-IIa,aac(6')-Ib3,aph(6)-Id,aph(3'')-Ib,aph(3')*	bla_{OXA-10},bla_{VIM-24}	*qnrVC6*	*msr(E), mph(E)*	/	*ARR-3*	*sul1*	/	*tmexCD1-TOprJ1*

										-VI,aadA1								
	30	*P. putida* ZXPA-20	ST236	pZXPA-20-602K	CP061724	Inc$_{pJBCL41}$	602,772	2019, Zhejiang, feces of migratory birds (28)	TSD8 (GACTGATC)	*aac(6')-Ib3,aph(6)-Id ,aph(3'')-Ib,aadA1*	*bla*$_{VIM-2}$	*qnrVC6*	/	/	*ARR-3*	*sul1*	*dfrB1*	*tet(C), tmexCD1-TOprJ1*
Group E (n=3)	31	*P. putida* MT178	ST300	pTmex-577K	CP143525	Inc$_{pJBCL41}$	577,576	2023, Shandong (29)	TSD7 (CAAGGGTC)	*aac(6')-Ib3,aac(6')-II aaac(6')-IIc aadA1*	*bla*$_{OXA-10}$,*bla*$_{VIM-2}$	*qnrVC6*	*msr(E), mph(E)*	/	*ARR-3*	*sul1*	*dfrA1 dfrB1*	*tmexCD1-TOprJ1*
	32	*P. putida* NY5709	ST78	pNY5709-IMP	MN961670	Inc$_{pJBCL41}$	636,818	2015, Hunan, urine (30)	TSD7 (CAAGGGTC)	*aac(6')-IIc,aac(6')-Ib3,aph(3'')-Ib,aph(6)-Id,aadA1*	*bla*$_{OXA-10}$,*bla*$_{VIM-2}$, *bla*$_{IMP-26}$	*qnrVC6*	*msr(E), mph(E)*	/	*ARR-3*	*sul1*	*dfrB1*	/
	33	*P. putida* NY11382	ST128	pNY11382-IMP	CP097104	Inc$_{pJBCL41}$	480,951	2011, Beijing, Homo sapiens (30)	TSD7 (CAAGGGTC)	*aac(6')-IIc,aph(6)-Id ,aph(3'')-Ib,aadA5*	*bla*$_{OXA-10}$, *bla*$_{IMP-34}$	/	/	/	/	*sul1*	/	*tmexCD1-TOprJ1*
Group F (n=1)	34	*P. aeruginosa* 1160	NA	p1160-VIM	MF144194	IncP-7β	205,426	2015, Hebei (32)	TSD5 (TTTCGATG)	*aac(6')-Ib3,aph(6)-Id ,aph(3'')-Ib,aadA1*	*bla*$_{VIM-24}$	*qnrVC6*	*msr(E), mph(E), ere(A)*	*catB3*	/	*sul1*	/	/
Group G (n=1)	35	*P. aeruginosa* AR19438	ST671	pAR19438/plasmid unnamed3	CP095924	Inc$_{pSTY-1}$	275,369	2021, Zhejiang (31)	CGCCGGGT	/	*bla*$_{AFM-4}$	*qnrVC6*	/	/	/	*sul1*	/	/

*NA, not available

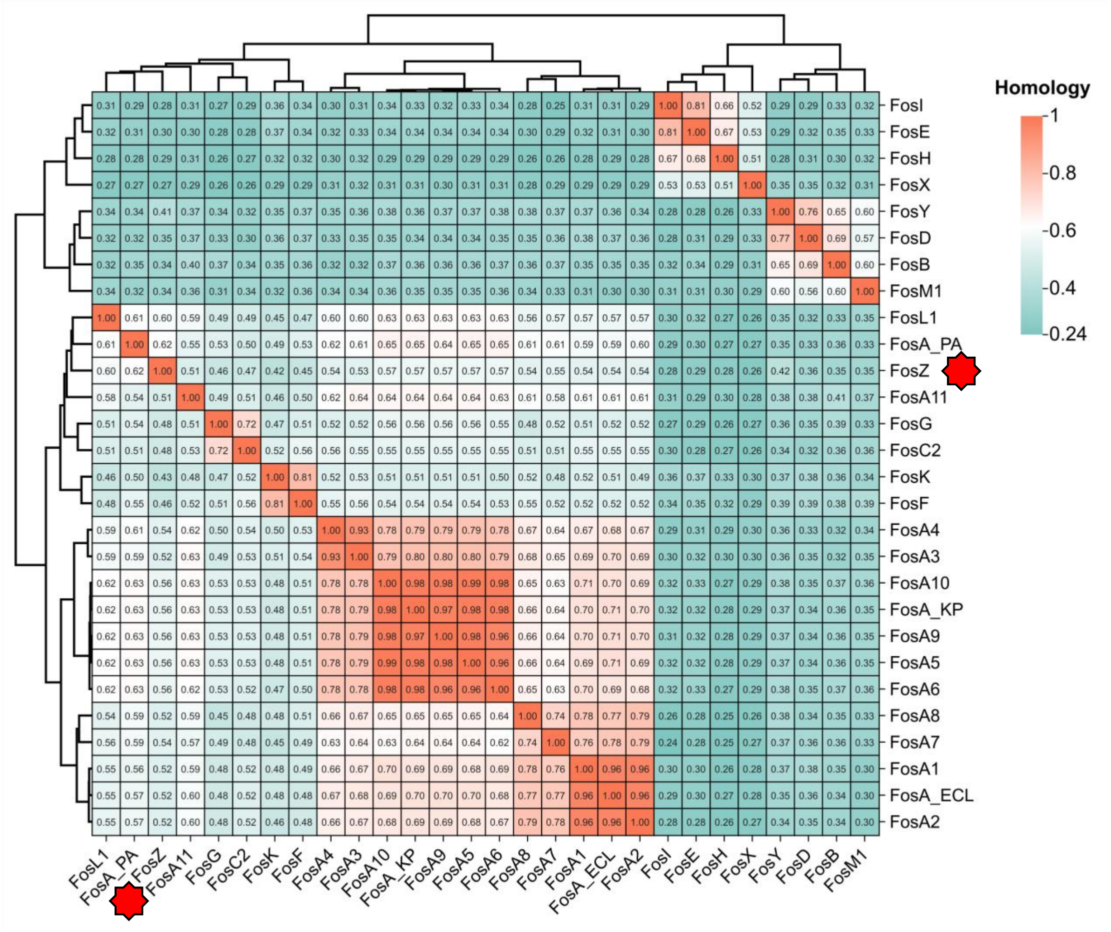

Figure S1. Amino Acid Homology of FosZ with Other FR-GSTs.

The numbers within the boxes indicate the degree of amino acid homology between the two proteins. FosZ (labeled with the red star symbol) exhibited the highest amino acid homology with $FosA^{PA}$ (62%) and FosL1 (60%).

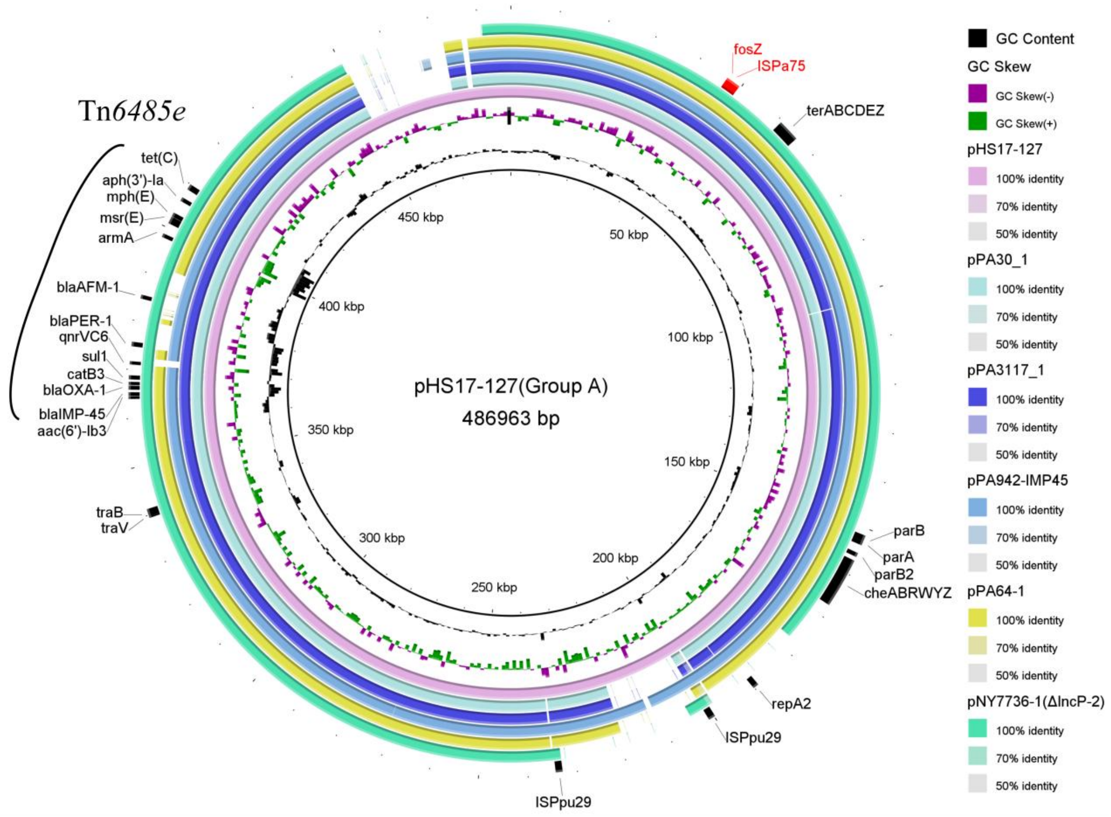

Figure S2. Circular Representation of Six Group A *fosZ*-bearing IncP-2 plasmids.

The innermost circle represents the pHS17-127 reference sequence, which contains a 57.3 kb multidrug resistance (MDR) transposon, Tn*6485e* (indicated by the black bracket). Typical IncP-2 plasmid modules in *Pseudomonas* species (21, 22) are highlighted as black arcs in the circular map, including the *repA2* gene encoding the IncP-2 replication initiator protein, stability (*parAB* and *parB2*), transfer systems, a tellurite resistance operon (*terABCDEZ*), and a chemotaxis operon (*cheABRWYZ*). IS*Pa75_fosZ* is highlighted as a red arc. The outermost circle represents the disabled IncP-2 (ΔIncP-2) plasmid pNY7736-1, caused by ISPpu29-mediated recombination , resulting about 22-kb fragment deletion around the repA2 replication gene. The circular map was constructed using BRIG.

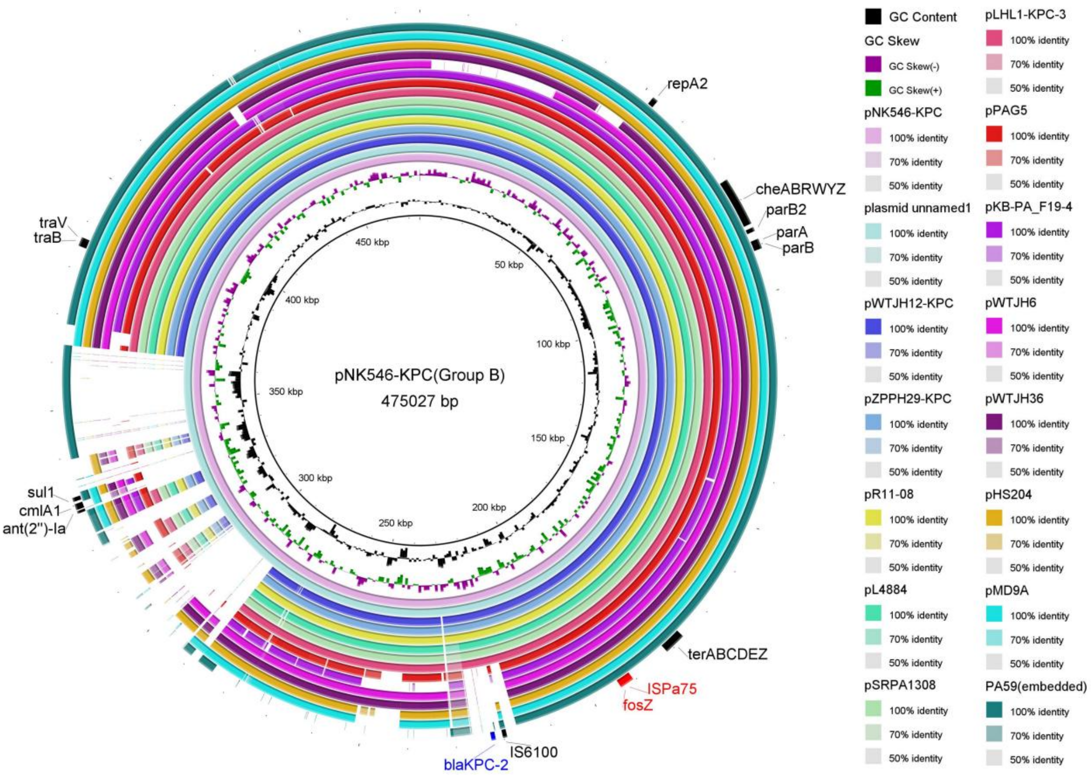

Figure S3. Circular Representation of 15 Groups B *fosZ*-bearing IncP-2 plasmids*.

The innermost circle represents the bla_{KPC-2}-containing pNK546-KPC reference sequence. Typical IncP-2 plasmid modules in *Pseudomonas* species are highlighted as black arcs in the circular map. IS*Pa75*_fosZ is highlighted as a red arc. The outermost circle represents the embedded IncP-2 plasmid located at positions nt 5239675-5678768 (439, 094 bp) in the chromosome of *P. aeruginosa* strain 59 at positions nt 5239675-5678768 (439, 094 bp).

*The nucleotide sequences of pSRPA1308 (CP158571) and plasmid unnamed 2 (OL468012) are identical. Consequently, only pSRPA1308 is presented.

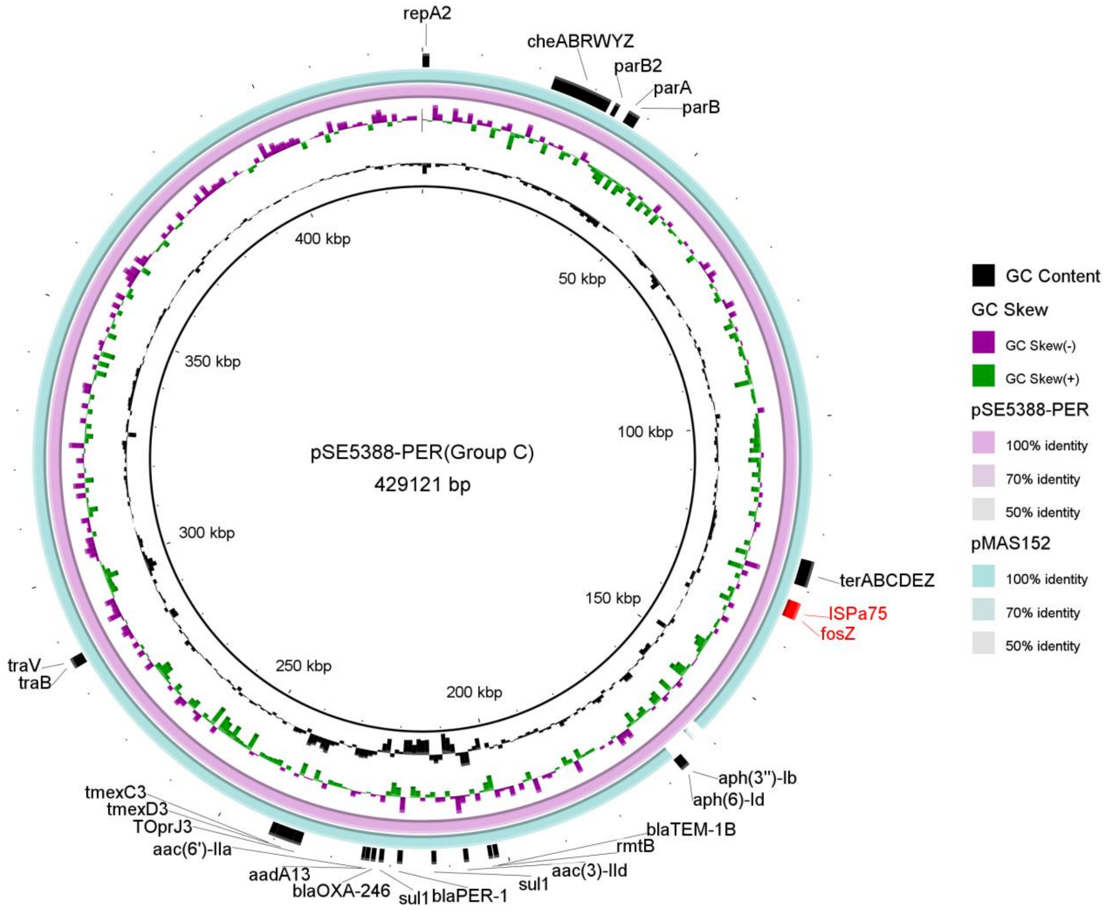

Figure S4. Circular Representation of two Group C *fosZ*-bearing IncP-2 plasmids.

The innermost circle represents the pSE5388-PER reference sequence. Typical IncP-2 plasmid modules in *Pseudomonas* species are highlighted as black arcs. IS*Pa75_fosZ* is highlighted as a red arc.

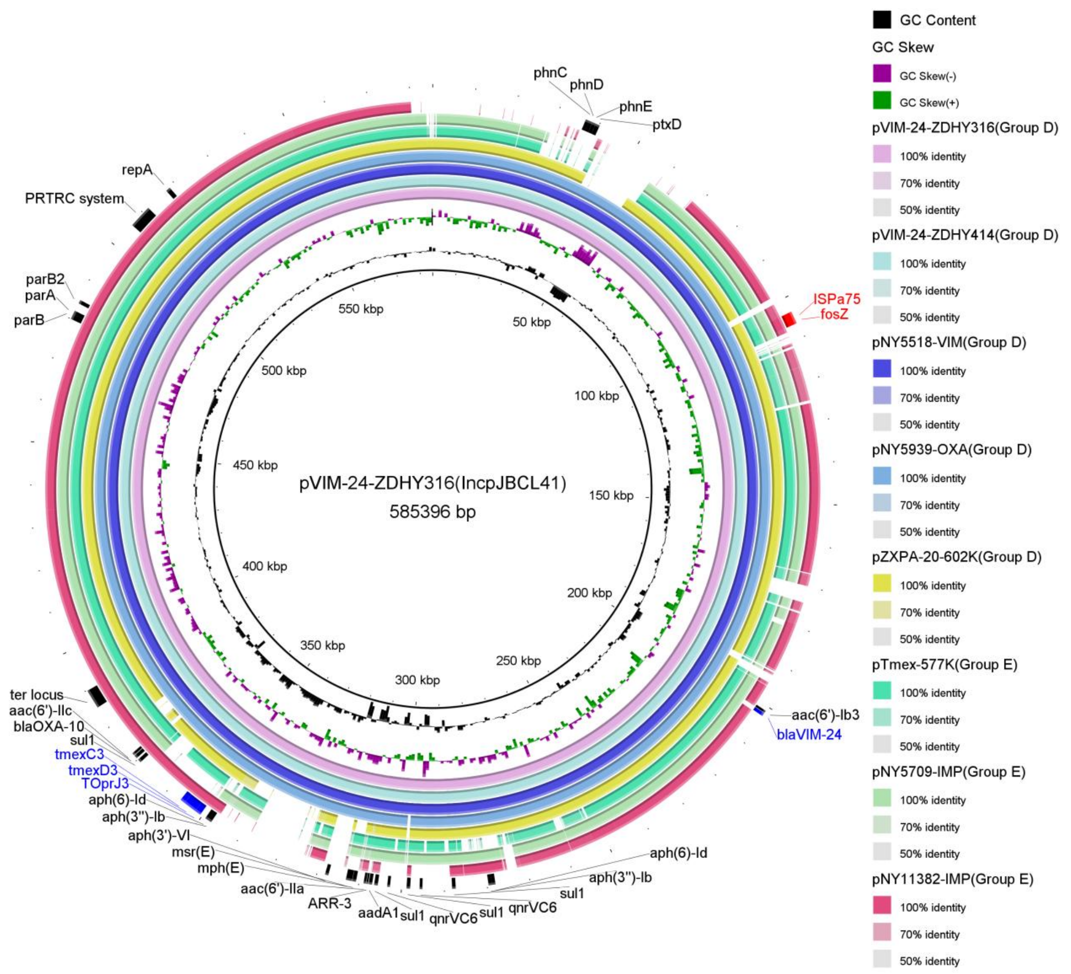

Figure S5. Circular Representation of eight Group D and Group E *fosZ*-bearing Inc$_{\mathrm{pJBCL41}}$ megaplasmids.

The innermost circle represents the pVIM-24-ZDHY316 reference sequence. Inc$_{\mathrm{pJBCL41}}$ megaplasmids modules are highlighted as black arcs in the circular map, including the replication initiator gene *repA*, the PRTRC system, stability (*parAB* and *parB2*), a tellurite resistance operon (*ter* loucs), and *phnCDE* encodes a phosphonate ABC transporter. IS*Pa75_fosZ* is highlighted as a red arc.

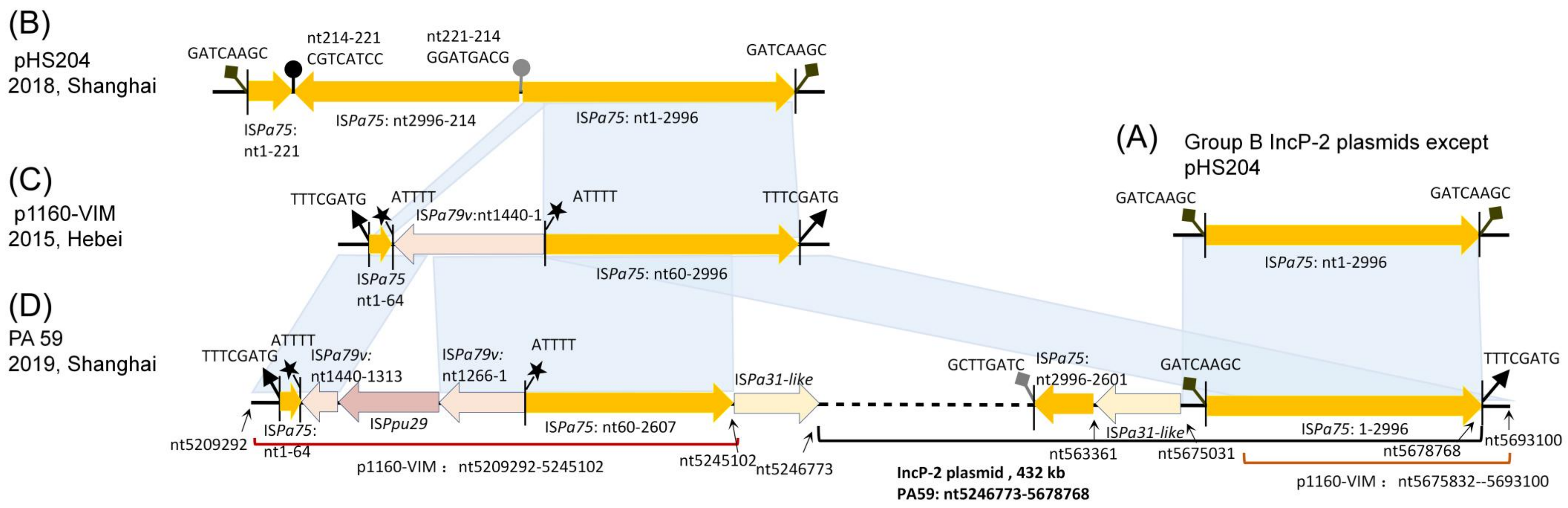

Figure S6. Evolution of IS*Pa75*-TSDs on IncP-2, IncP-7β plasmids and a chromosome of *Pseudomonas aeruginosa*.

(A) IS*Pa75*-TSD6 on Group B IncP-2 plasmids except pHS204. (B) IS*Pa75*-TSD6 on Group B plasmid pHS204 with tandem repeats of IS*Pa75* and partial of one IS*Pa75* inverted (nt214-2996), (C) IS*Pa75*-TSD5 on IncP-7β plasmid p1160-VIM which disrupted by IS*Pa79* at nt60-64, and (D) IS*Pa75*-TSD5/TSD6 on the chromosome of *Pseudomonas aeruginosa* strain 59, which recruited an IncP-2 plasmid (nt5246773-5678768) and partial segments of p1160-VIM (nt5209292-5245102, 5675832-5693100). IS*Pa75* is bounded by left and right inverted repeats (IR_L/IR_R) and flanked by 8-bp target sequence duplications (TSDs, TSD6-GATCAAGC or TSD5-TTTCGATG), as indicated by paired filled squares or arrows.

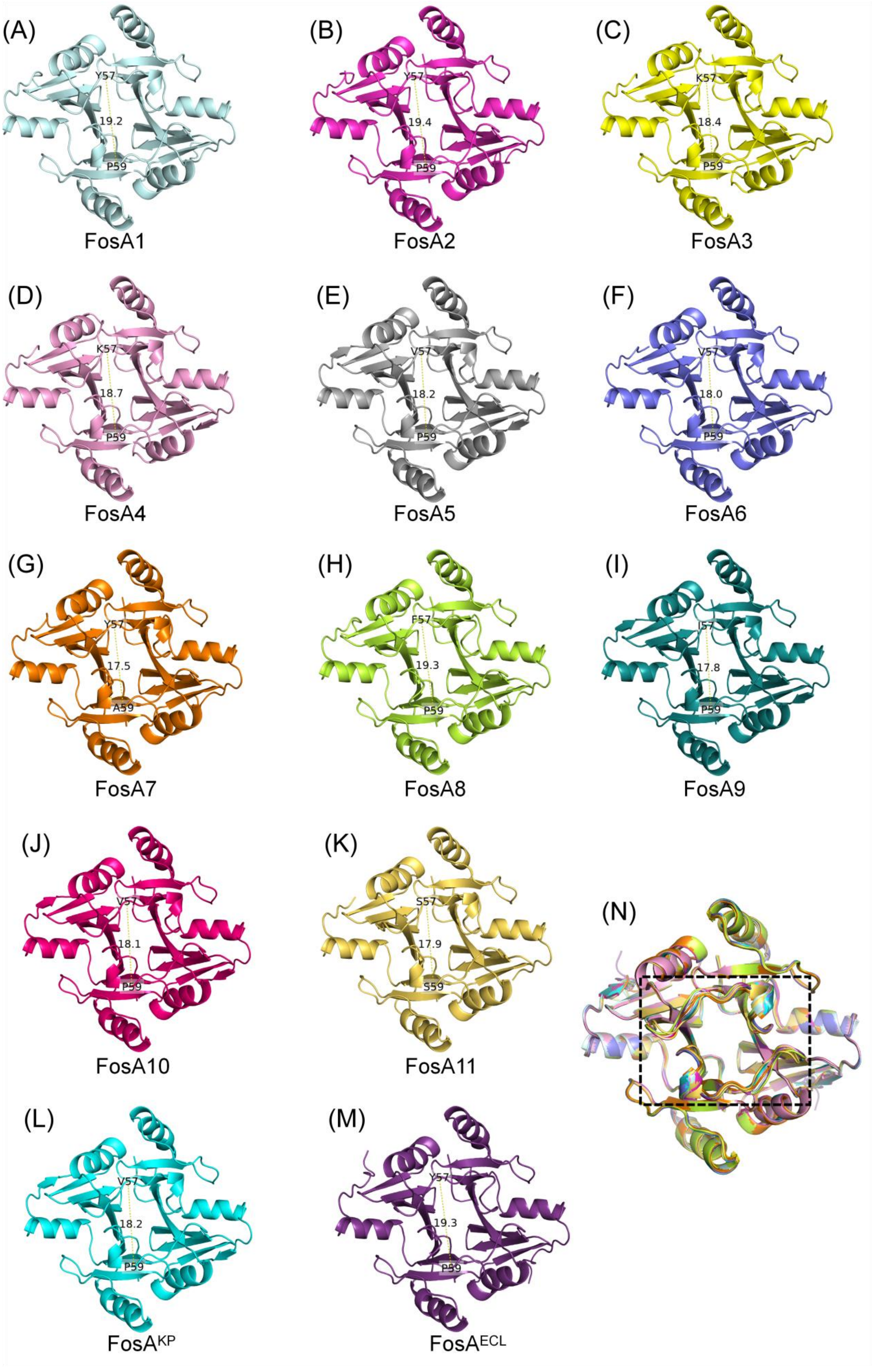

Figure S7. Predicted protein structures of FosA-family members (FosA1 to FosA11, FosA^{KP}, and FosA^{ECL}) using ColabFold.

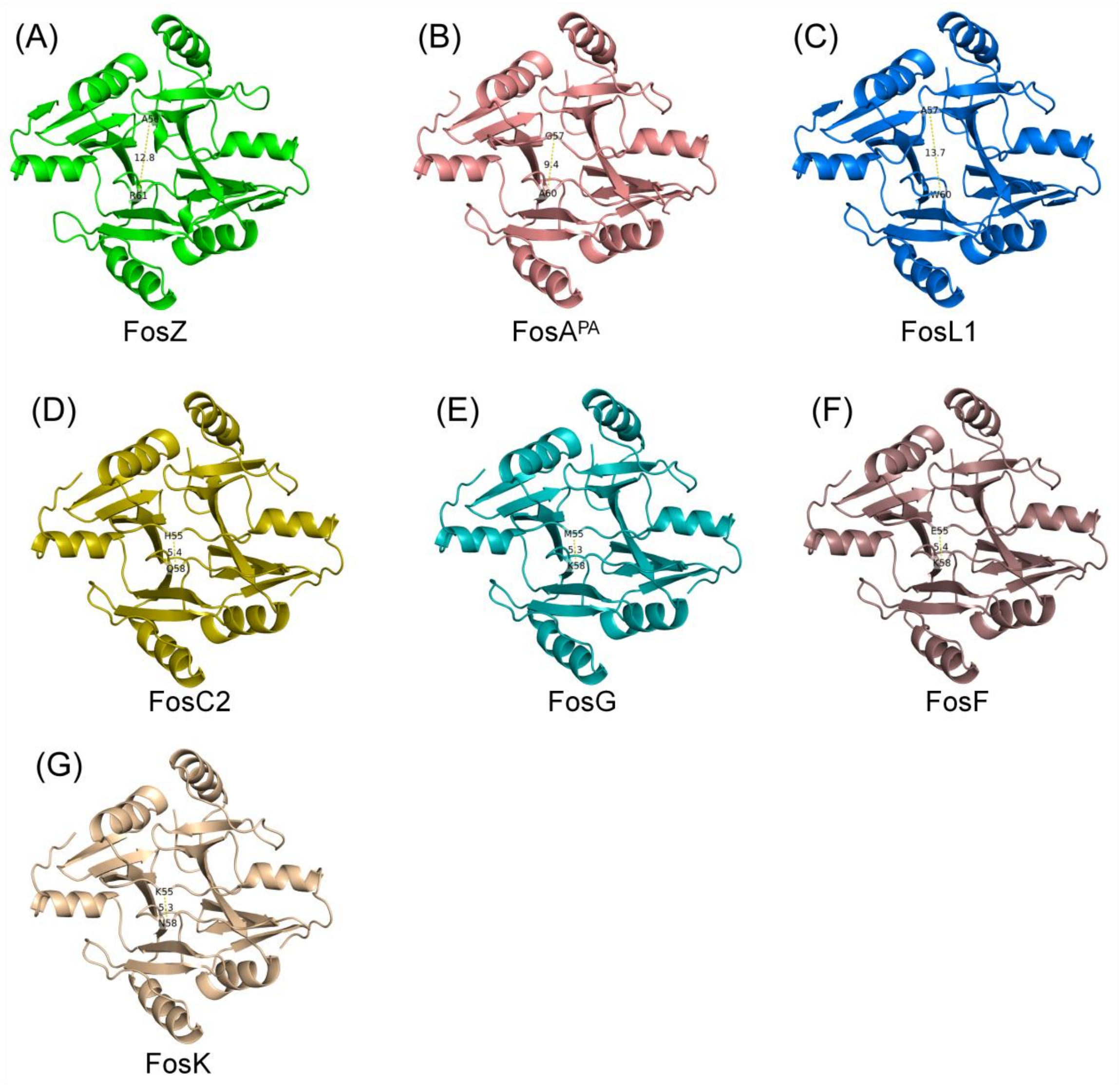

Figure S8. Predicted protein structures of FR-GSTs (FosZ, FosA^{PA}, FosL1, FosC2, FosG, FosF, and FosK) using ColabFold

Reference

(1) Navas J, León J, Arroyo M, García Lobo JM. Nucleotide sequence and intracellular location of the product of the fosfomycin resistance gene from transposon Tn2921. Antimicrob Agents Chemother. 1990 Oct;34(10):2016-8.

(2) Xu H, Miao V, Kwong W, Xia R, Davies J. Identification of a novel fosfomycin resistance gene (*fosA2*) in Enterobacter cloacae from the Salmon River, Canada. Lett Appl Microbiol. 2011 Apr;52(4):427-9.

(3) Wachino J, Yamane K, Suzuki S, Kimura K, Arakawa Y. Prevalence of fosfomycin resistance among CTX-M-producing *Escherichia coli* clinical isolates in Japan and identification of novel plasmid-mediated fosfomycin-modifying enzymes. Antimicrob Agents Chemother. 2010 Jul;54(7):3061-4.

(4) Nakamura G, Wachino J, Sato N, Kimura K, Yamada K, Jin W, Shibayama K, Yagi T, Kawamura K, Arakawa Y. Practical agar-based disk potentiation test for detection of fosfomycin-nonsusceptible *Escherichia coli* clinical isolates producing glutathione S-transferases. J Clin Microbiol. 2014 Sep;52(9):3175-9.

(5) Ma Y, Xu X, Guo Q, Wang P, Wang W, Wang M. Characterization of *fosA5*, a new plasmid-mediated fosfomycin resistance gene in *Escherichia coli*. Lett Appl Microbiol. 2015 Mar;60(3):259-64.

(6) Guo Q, Tomich AD, McElheny CL, Cooper VS, Stoesser N, Wang M, Sluis-Cremer N, Doi Y. Glutathione-S-transferase FosA6 of *Klebsiella pneumoniae* origin conferring fosfomycin resistance in ESBL-producing *Escherichia coli*. J Antimicrob Chemother. 2016 Sep;71(9):2460-5.

(7) Rehman MA, Yin X, Persaud-Lachhman MG, Diarra MS. First Detection of a Fosfomycin Resistance Gene, *fosA7*, in *Salmonella enterica* Serovar Heidelberg Isolated from Broiler Chickens. Antimicrob Agents Chemother. 2017 Jul 25;61(8):e00410-17.

(8) Poirel L, Vuillemin X, Kieffer N, Mueller L, Descombes MC, Nordmann P. Identification of FosA8, a Plasmid-Encoded Fosfomycin Resistance Determinant from *Escherichia coli*, and Its Origin in *Leclercia adecarboxylata*. Antimicrob Agents Chemother. 2019 Oct 22;63(11):e01403-19.

(9) Ten Doesschate T, Abbott IJ, Willems RJL, Top J, Rogers MRC, Bonten MM, Paganelli FL. *In vivo* acquisition of fosfomycin resistance in *Escherichia coli* by *fosA* transmission from commensal flora. J Antimicrob Chemother. 2019 Dec 1;74(12):3630-3632.

(10) Huang Y, Lin Q, Zhou Q, Lv L, Wan M, Gao X, Wang C, Liu JH. Identification of *fosA10*, a Novel Plasmid-Mediated Fosfomycin Resistance Gene of *Klebsiella pneumoniae* Origin, in *Escherichia coli*. Infect Drug Resist. 2020 May 1;13:1273-1279.

(11) Lu W, Zhou S, Ma X, Xu N, Liu D, Zhang K, Zheng Y, Wu S. *fosA11*, a novel chromosomal-encoded fosfomycin resistance gene identified in *Providencia rettgeri*. Microbiol Spectr. 2024 Feb 6;12(2):e0254223.

(12) Yatsuyanagi J, Saito S, Harata S, Suzuki N, Ito Y, Amano K, Enomoto K. Class 1 integron containing metallo-beta-lactamase gene $bla_{\text{VIM-2}}$ in *Pseudomonas aeruginosa* clinical strains isolated in Japan. Antimicrob Agents Chemother. 2004 Feb;48(2):626-8.

(13) Kieffer N, Poirel L, Descombes MC, Nordmann P. Characterization of FosL1, a Plasmid-Encoded Fosfomycin Resistance Protein Identified in *Escherichia coli*. Antimicrob Agents Chemother. 2020 Mar 24;64(4):e02042-19.

(14) Zheng D, Bergen PJ, Landersdorfer CB, Hirsch EB. Differences in Fosfomycin Resistance Mechanisms between *Pseudomonas aeruginosa* and *Enterobacterales*. Antimicrob Agents Chemother. 2022 Feb 15;66(2):e0144621.
(15) Kitanaka H, Wachino J, Jin W, Yokoyama S, Sasano MA, Hori M, Yamada K, Kimura K, Arakawa Y. Novel integron-mediated fosfomycin resistance gene *fosK*. Antimicrob Agents Chemother. 2014 Aug;58(8):4978-9.
(16) Zilhao R, Courvalin P. Nucleotide sequence of the *fosB* gene conferring fosfomycin resistance in *Staphylococcus epidermidis*. FEMS Microbiol Lett. 1990 Mar 15;56(3):267-72.
(17) Pelegrino Kde O, Campos JC, Sampaio SC, Lezirovitz K, Seco BM, Pereira Mde O, Rocha DA, Jové T, Nicodemo AC, Sampaio JL. *fosI* Is a New Integron-Associated Gene Cassette Encoding Reduced Susceptibility to Fosfomycin. Antimicrob Agents Chemother. 2015 Nov 9;60(1):686-8.
(18) Khabthani S, Hamel M, Baron SA, Diene SM, Rolain JM, Merhej V. *fosM*, a New Family of Fosfomycin Resistance Genes Identified in Bacterial Species Isolated from Human Microbiota. Antimicrob Agents Chemother. 2021 Jan 20;65(2):e01712-20.
(19) Chen Y, Ji S, Sun L, Wang H, Zhu F, Chen M, Zhuang H, Wang Z, Jiang S, Yu Y, Chen Y. The novel fosfomycin resistance gene *fosY* is present on a genomic island in CC1 methicillin-resistant *Staphylococcus aureus*. Emerg Microbes Infect. 2022 Dec;11(1):1166-1173.
(20) Fillgrove KL, Pakhomova S, Newcomer ME, Armstrong RN. Mechanistic diversity of fosfomycin resistance in pathogenic microorganisms. J Am Chem Soc. 2003 Dec 24;125(51):15730-1.
(21) Zhang X, Wang L, Li D, Wang C, Guo Q, Wang M. Characterization of the novel plasmid-encoded MBL gene *bla*$_{\mathrm{AFM-1}}$, integrated into a *bla*$_{\mathrm{IMP-45}}$-bearing transposon Tn6485e in a carbapenem-resistant *Pseudomonas aeruginosa* clinical isolate. J Antimicrob Chemother. 2021 Dec 24;77(1):83-8
(22) Zhang X, Wang L, Li D, Li P, Yuan L, Yang F, Guo Q, Wang M. An IncP-2 plasmid sublineage associated with dissemination of *bla*$_{\mathrm{IMP-45}}$ among carbapenem-resistant *Pseudomonas aeruginosa*. Emerg Microbes Infect. 2021 Dec;10(1):442-449.
(23) Fang Y, Wang N, Wu Z, Zhu Y, Ma Y, Li Y, Cai H, Zhang P, Leptihn S, Yu Y, Hua X, Tu Y. An XDR *Pseudomonas aeruginosa* ST463 Strain with an IncP-2 Plasmid Containing a Novel Transposon Tn6485f Encoding *bla*$_{\mathrm{IMP-45}}$ and *bla*$_{\mathrm{AFM-1}}$ and a Second Plasmid with Two Copies of *bla*$_{\mathrm{KPC-2}}$. Microbiol Spectr. 2023 Feb 14;11(1):e0446222.
(24) Zhou L, Yang C, Zhang X, Yao J, Chen L, Tu Y, Li X. Characterization of a novel Tn6485h transposon carrying both *bla*$_{\mathrm{IMP-45}}$ and *bla*$_{\mathrm{AFM-1}}$ integrated into the IncP-2 plasmid in a carbapenem-resistant *Pseudomonas aeruginosa*. J Glob Antimicrob Resist. 2023 Dec;35:307-313.
(25) Zhang Y, Meng L, Guan C, Zhou Y, Peng J, Liang H. Genomic characterisation of clinical *Pseudomonas aeruginosa* isolate PAG5 with a multidrug-resistant megaplasmid from China. J Glob Antimicrob Resist. 2020 Jun;21:130-131.
(26) Long X, Zhang H, Wang X, Mao D, Wu W, Luo Y. RecT Affects Prophage Lifestyle and Host Core Cellular Processes in *Pseudomonas aeruginosa*. Appl Environ Microbiol. 2022 Sep 22;88(18):e0106822.
(27) Wang L, Zhang X, Zhou X, Bi Y, Wang M, Guo Q, Yang F. Insertion of ISPa1635 in ISCR1

Creates a Hybrid Promoter for *bla*$_{PER-1}$ Resulting in Resistance to Novel β-lactam/β-lactamase Inhibitor Combinations and Cefiderocol. Antimicrob Agents Chemother. 2023 Jun 15;67(6):e0013523.

(28) Li R, Peng K, Xiao X, Liu Y, Peng D, Wang Z. Emergence of a multidrug resistance efflux pump with carbapenem resistance gene *bla*$_{VIM-2}$ in a *Pseudomonas putida* megaplasmid of migratory bird origin. J Antimicrob Chemother. 2021 May 12;76(6):1455-1458.

(29) Long X, Li J, Yang H, Gao Y, Zeng X, Tang B. 2024. Discovery and characterization of *tmexCD3-toprJ1* on a plasmid from *Pseudomonas putida* isolated in a public trash can. Microbiol Spectr 12:e00395-24.

(30) Li X, Mu X, Chen F, Lu X, He J, Zheng Y, Zhou D, Yin Z, Wang P. Characterization of Three Novel IMP Metallo-β-Lactamases, IMP-89, IMP-91, and IMP-96, and Diverse *bla*$_{IMP}$-Carrying Accessory Genetic Elements from Chinese Clinical Isolates. Microbiol Spectr. 2023 Jun 15;11(3):e0498622.

(31) Chen M, Cai H, Li Y, Wang N, Zhang P, Hua X, Yu Y, Sun R. Plasmid-Borne AFM Alleles in *Pseudomonas aeruginosa* Clinical Isolates from China. Microbiol Spectr. 2022 Oct 26;10(5):e0203522.

(32) Zeng L, Zhan Z, Hu L, Jiang X, Zhang Y, Feng J, Gao B, Zhao Y, Yang W, Yang H, Yin Z, Zhou D. Genetic Characterization of a *bla*$_{VIM-24}$-Carrying IncP-7β Plasmid p1160-VIM and a *bla*$_{VIM-4}$-Harboring Integrative and Conjugative Element Tn6413 From Clinical *Pseudomonas aeruginosa*. Front Microbiol. 2019 Feb 26;10:213
